# Smoking, Exhaled Carbon Monoxide, and Risk of Parkinson Disease

**DOI:** 10.1001/jamaneurol.2026.3037

**Published:** 2026-09-08

**Authors:** Clara Bueno Lopez, Andri Iona, Iain Turnbull, Huaidong Du, Yiping Chen, Ling Yang, Alexander Tinworth, Zhengjie Shen, Kshitij Kolhe, Pei Pei, Canqing Yu, Dianjianyi Sun, Jun Lv, Junshi Chen, Robert Clarke, Rory Collins, Richard Peto, Liming Li, Fiona Bragg, Zhengming Chen

**Affiliations:** 1 Clinical Trial Service Unit & Epidemiological Studies Unit, Nuffield Department of Population Health, University of Oxford, Oxford, United Kingdom; 2 Noncommunicable Diseases Prevention and Control Department, Tongxiang Center for Disease Control and Prevention, Tongxiang, China; 3 Peking University Center for Public Health and Epidemic Preparedness & Response, Beijing, China; 4 Department of Epidemiology and Biostatistics, School of Public Health, Peking University Health Science Center, Beijing, China; 5 Key Laboratory of Epidemiology of Major Diseases, Peking University, Ministry of Education, Beijing, China; 6 China National Center for Food Safety Risk Assessment, Chaoyang District, Beijing, China; 7 Health Data Research UK Oxford, University of Oxford, Oxford, United Kingdom

## Abstract

**Importance:**

Smoking has been associated with lower risks of Parkinson disease (PD) in epidemiological studies, but the underlying mechanisms remain poorly elucidated.

**Objective:**

To investigate the association of exhaled carbon monoxide (CO) with risk of PD.

**Design, Setting, and Participants:**

This nationwide prospective cohort study leveraged data from the China Kadoorie Biobank across 10 geographically diverse areas in China. A total of 512 724 adults aged 30 to 79 years were recruited between June 2004 and July 2008. Prospective analyses were restricted to first events occurring between ages 40 and 94 years and before January 1, 2019. Data were analyzed from May 2025 to March 2026.

**Exposure:**

Self-reported smoking status and measured exhaled CO.

**Main Outcomes and Measures:**

During approximately 12 years’ follow-up, 1131 individuals with PD and 2949 with other neurodegenerative diseases were recorded through linkage to national death and disease registries and the health insurance system. Cox regression was used to estimate adjusted hazard ratios (HRs) for associations of smoking and, among individuals who never smoked, exhaled CO levels with PD, other neurodegenerative diseases, and several known smoking-related diseases. Covariates included sociodemographic characteristics, lifestyle factors, and other confounders (ie, solid fuel use and passive exposure to smoking).

**Results:**

Among the 512 701 participants included in the main analyses, the mean (SD) age was 52 (11) years and 302 041 (58.9%) were female. Overall, 156 650 male individuals (74.5%) and 9944 female individuals (3.3%) ever smoked regularly, with mean exhaled CO levels higher among those who regularly smoked (11.1 ppm) than those who never smoked (3.5 ppm), those who occasionally smoked (3.8 ppm), and those who formerly smoked regularly (3.7 ppm). Regular smoking was significantly associated with elevated risks of lung cancer, ischemic heart disease, stroke, and all-cause mortality but with lower risk of PD (adjusted HR, 0.70; 95% CI, 0.62-0.79). In individuals who never smoked, higher exhaled CO levels were associated with lower HRs of PD (n = 675) in a broadly dose-dependent manner (exhaled CO <2.0 ppm: 1.00 [95% CI, 0.85-1.18]; 2.0 to <3.0: 0.85 [95% CI, 0.73-0.99]; 3.0 to <5.0: 0.62 [95% CI, 0.53-0.73]; 5.0 to <11.5: 0.68 [95% CI, 0.56-0.83]; ≥11.5: 0.65 [95% CI, 0.45-0.92]; *P* for trend < .001) but were not associated with risks of smoking-related diseases or other neurodegenerative diseases. The associations with PD were more pronounced in female individuals who never smoked. Similar exhaled CO-PD associations were found in those who regularly smoked.

**Conclusion and Relevance:**

Among individuals who never smoked, higher exhaled CO levels were associated with lower PD risk. These findings demonstrate a potentially protective role of CO in PD etiology, which help explain the association between smoking and PD observed in this and other epidemiological studies and support ongoing clinical trials of CO in the treatment of PD.

## Key Points

**Question:**

Could carbon monoxide (CO) explain the association between smoking and lower risk of Parkinson disease (PD)?

**Findings:**

In this cohort study of 512 701 Chinese adults, regular smoking was associated with a lower risk of PD. Among individuals who never smoked, higher exhaled CO levels were associated with lower PD risk but not with risk of other smoking-related diseases.

**Meaning:**

These findings suggest that the inverse association between smoking and PD may not be attributable to tobacco smoking itself and provide evidence that CO exposure may contribute to this association.

## Teaser

This cohort study assesses prospective associations between carbon monoxide exhalation and lower risk of Parkinson disease.

## Introduction

Parkinson disease (PD) is the second most common neurodegenerative condition worldwide, affecting more than 10 million adults globally.^1,2^ While the etiology of PD remains incompletely understood, epidemiological studies in diverse populations have repeatedly documented lower risks of PD among individuals who smoke,^3,4,5,6,7^ typically displaying a dose-response association with smoking intensity and duration.^3,8,9,10^ In preclinical studies, cigarette smoking conferred protection in rodent toxin models of PD, providing support for the biological plausibility of the observed association in epidemiological studies, with nicotine hypothesized as a possible protective compound.^3,4^ However, a recent double-blind randomized clinical trial showed no benefit of 1-year transdermal nicotine therapy on motor function and clinical progression (measured by the Unified Parkinson’s Disease Rating Scale) in patients with treatment-naive PD and, if anything, there appeared to be a tendency toward worsening of total mean Unified Parkinsons Disease Rating Scale score in the treatment group.^11^ Among the thousands of other chemicals and gases present in cigarette smoke,^12^ a few (eg, hydroquinone and compounds acting as monoamine oxidase-B inhibitors) have been proposed that may underlie the smoking-PD association.^3,4^ However, supporting evidence is lacking for a role for any of these in PD etiology.

Carbon monoxide (CO) is a well-known by-product of smoking^13,14,15^ and other environmental exposures (eg, solid fuel use). While exposure to high levels of environmental CO (eg, gas poisoning) has severe adverse health consequences,^16,17^ research on the long-term effects of exposure to lower, nonlethal concentrations of CO has largely coupled it to harmful effects in the context of smoking and air pollution.^15,18,19^ However, CO is also an endogenously produced essential signaling molecule that provides protection against oxidative damage and supports various physiological functions, including immune response and cell proliferation, survival, and death.^20,21,22,23^ Exposure to low concentrations of CO has shown neuroprotective effects in vitro,^24,25,26,27^ and animal experiments have also suggested a protective role in PD.^28,29^ This has led to new development efforts for the treatment of PD through targeting CO (NCT07005180). However, there is currently no evidence from human epidemiological and experimental studies supporting the role of CO in PD etiology. Although previous epidemiological studies have investigated the associations of smoking and air pollution^30,31^ (2 leading sources of environmental CO) with PD, these exposures are inevitably confounded by the effects of other gases, particles, and chemicals with well-known adverse health consequences.

The present study presents detailed analyses of smoking and exhaled CO with risks of PD and other neurodegenerative diseases in the prospective China Kadoorie Biobank (CKB). Specifically, we aimed to clarify the association of exhaled CO with risk of PD in individuals who never smoked while controlling for other known environmental sources of CO.

## Methods

### Study Population

The CKB study design and participants have been previously described.^32,33^ Briefly, 512 724 adults predominantly aged 35 to 79 years, including a small number outside this target age range, from 10 (5 urban and 5 rural) geographically defined areas of China were recruited between June 2004 and July 2008. All eligible adults without major disability from carefully selected local communities of each study area were invited to participate. Ethics approval was obtained from the Oxford University Tropical Research Ethics Committee and the Chinese Center for Disease Control and Prevention Ethical Review Committee, and all participants provided written informed consent. This study followed the Strengthening the Reporting of Observational Studies in Epidemiology (STROBE) reporting guideline.

Trained health workers administered laptop-based questionnaires, collecting detailed information on sociodemographic characteristics, lifestyle (eg, smoking, alcohol drinking, diet, and physical activity), living conditions (eg, heating and cooking fuel types and passive exposure to smoking), and medical history. Trained technicians also took various physical measurements (eg, height, weight, blood pressure, lung function, and exhaled CO) using calibrated instruments and standardized protocols.^33^

### Smoking and Exhaled CO

At the baseline survey, detailed information on smoking exposure was collected, including usual smoking status. For those who reported ever regularly smoking, further information was collected on the frequency, type and amount of tobacco smoked, age at smoking initiation, level of inhalation, cigarettes smoked (or equivalent other tobacco product) on the day of the survey, smoking duration (in years), and reason for smoking cessation (as applicable). Participants were classified as those who regularly smoked (those who reported smoking daily or almost every day), those who previously smoked regularly (those who had ceased smoking regularly for at least 6 months), those who occasionally smoked (if they did not meet the criteria for regular smoking), and those who never smoked.

Exhaled CO was measured using a MicroCO meter (CareFusion) in accordance with the manufacturer’s instructions, with regular calibrations following standard protocol. Participants were asked to inhale deeply and hold their breath for 20 seconds (or no less than 10 seconds due to frailty in some cases) before exhaling slowly into the mouthpiece of the calibrated meter. Two measures were taken and the mean value of the 2 readings was used in analyses.^34,35^ The device additionally provided an estimation of blood carboxyhemoglobin using previously documented mathematical relationships^14,36^ between exhaled CO and carboxyhemoglobin (eTable 1 in Supplement 1).

### Morbidity and Mortality Follow-Up

The vital status of participants was monitored regularly through linkage, using unique 18-digit national identification numbers, to local death registries, supplemented by annual active confirmation through local residential, health insurance, and administrative records. Additional information on morbidity was collected through linkage with established disease registries (for cancer, stroke, ischemic heart disease, and diabetes) and the national health insurance system, which records any episodes of hospitalization with almost universal (>98%) coverage of all residents in the study areas. In addition, the very small number of participants not covered by the health insurance system were actively followed up on an annual basis via telephone and home visits collecting information on hospitalization and major health outcomes and ensuring completeness of follow-up for these participants. All events were coded with *International Classification of Diseases, 10th revision* (*ICD-10*), codes by trained staff who were blinded to participants’ baseline information. By January 1, 2019, a total of 56 549 participants (11.0%) had died and 4028 (<1%) were lost to follow-up.

The main outcomes analyzed in the present study were PD (n = 1131) and other neurodegenerative diseases without a codiagnosis of parkinsonism (dementia, brain atrophy, and macular degeneration [n = 2949]). Several smoking-related diseases (lung cancer, ischemic heart disease, and stroke) and all-cause mortality were also included as positive controls to assess the specificity of the associations. The recorded numbers and associated *ICD-10* codes of these outcomes are detailed in eTable 2 in Supplement 1. Analyses were restricted to first events between the ages of 40 and 94 years and prior to January 1, 2019, with censoring at the event of interest, death, or loss to follow-up, whichever occurred first.

### Statistical Analyses

Participants with extreme values of exhaled CO (outside the range of 1-99 ppm [n = 21]) or missing body mass index data (n = 2) were excluded, leaving 512 701 participants for inclusion in the main analyses.

Since the distribution of exhaled CO was positively skewed, adjusted geometric means and 95% CIs of exhaled CO concentrations by smoking status and number of cigarettes smoked were calculated using linear regression. Analyses were repeated separately among male individuals and female individuals, and urban and rural residents. Participant baseline characteristics were examined by exhaled CO quintiles (<2.0, 2.0 to <3.0, 3.0 to <5.0, 5.0 to <11.5, ≥11.5 ppm), with the prevalence or mean values standardized by 5-year age groups and sex, as appropriate, and by incident PD outcome, additionally standardized by study area.

Directly standardized incidence rates and 95% CIs for PD were calculated based on γ approximation and standardized by age at risk (5-year groups), sex, and study area, as appropriate. For prospective analyses, smoking was categorized as never regular (never and occasional smoking), former regular (former regular smoking stopped for reasons other than illness), and regular (regular smoking at baseline and those who stopped because of illness) (eFigure 1 in Supplement 1). Among those who ever smoked regularly, smoking pack-years was derived as the usual number of cigarettes per day divided by 20, multiplied by years smoked. Among individuals who never smoked, passive exposure to smoking was defined as having ever lived for at least 6 months with someone who smoked or being exposed to other people’s tobacco smoke (for a minimum of 5 consecutive minutes each time) at home, in the workplace, or public places. Adjusted hazard ratios (HRs) for specific diseases associated with smoking and exhaled CO (among individuals who never smoked) were estimated using Cox proportional hazard models, stratified by age at risk (5-year groups), sex, and study area (n = 10) and adjusted for education (no formal school, primary school, middle school, high school, technical school/college, and university), body mass index (calculated as weight in kilograms divided by height in meters squared), alcohol drinking (never/ever regular), and physical activity (metabolic equivalent of task hours per day). For analyses of exhaled CO among individuals who never smoked, additional adjustments for season, hour at survey, solid fuel use (yes/no), and passive exposure to smoking (yes/no) were also made. All covariates were selected a priori based on evidence from previous studies of their associations with exhaled CO, smoking, and PD. The 95% CIs were estimated using the variance of each group-specific log hazard, allowing comparisons between any 2 groups.^37^

In subgroup analyses of exhaled CO and PD, we used a cut point of 3 ppm (median exhaled CO in individuals who never smoked) to estimate HRs across different population subgroups (eg, study area, education, body mass index, and self-reported health), follow-up periods (<5, 5 to <10, and ≥10 years), and age-at-risk groups (<65, 65-74, and ≥75 years). We also undertook various sensitivity analyses, applying different exclusion criteria (eg, regular smoking at baseline; individuals with prior chronic diseases; and participants from Henan, a heavily polluted rural area in which most households at study recruitment used coal for cooking and heating in winter and did not have proper chimneys for cookstoves^38,39^) and models with different adjustments, using group-specific exhaled CO quintiles, restricting the PD case definition to participants with more than 1 recorded diagnosis of PD during study follow-up, and redefining the outcome to include any parkinsonism. All statistical analyses were done using R (version 4.4.3). Data were analyzed from May 2025 to March 2026.

## Results

Of the 512 701 participants included, the mean (SD) age at baseline was 52.0 (10.7) years, 302 041 individuals (58.9%) were female, 226 942 (44.3%) resided in urban areas, 187 835 (36.6%) used solid fuel for cooking, and 156 650 (74.5%) of male individuals and 9944 (3.3%) of female individuals reported ever regular smoking (Table). Participants with an incident PD diagnosis during follow-up were older at baseline (mean [SD] age, 61.3 [9.8] years) and a relatively lower proportion (584 [51.7%]) were female (eTable 3 in Supplement 1). Baseline exhaled CO levels were inversely associated with age and income; positively associated with regular alcohol drinking, regular smoking, and, in rural areas, with use of solid fuels for cooking; and showed no clear significant associations with prevalence of major chronic diseases. A larger proportion of participants in the highest one-fifth of exhaled CO levels (≥11.5 ppm), compared with lower exhaled CO levels, was recruited in winter (31 107 [29.9%] vs 19 011 [17.1%]-19 931 [23.0%]), and lived in rural areas (74 703 [71.7%] vs 53 462 [48.0%]-68 247 [58.1%]). These sociodemographic and environmental differences were explained almost entirely by differences in study area. Patterns were more pronounced in female individuals who never smoked, with qualitatively similar findings in male individuals who never smoked (eTable 4 in Supplement 1). Among individuals who never smoked, the observed urban-rural differences in exhaled CO levels were driven mainly by the inclusion of Henan, which contributed 67.1% of female (n = 13 821) and 44.1% of male (n = 957) individuals who never smoked in the highest exhaled CO quintile (eFigure 2 in Supplement 1).

**Table.** Baseline Characteristics of Study Participants by Exhaled Carbon Monoxide (CO) Level^a^

Characteristic	Exhaled CO, ppm					Total (N = 512 701)
	<2.0 (n = 86 680)	2.0 to <3.0 (n = 92 929)	3.0 to <5.0 (n = 111 393)	5.0 to <11.5 (n = 117 554)	≥11.5 (n = 104 145)	
Exhaled CO, mean (SD), ppm	1.2 (0.3)	2.2 (0.3)	3.6 (0.6)	7.2 (1.9)	20.8 (14.3)	7.3 (7.9)
Estimated COHb, mean (SD), %	0.2 (0.1)	0.4 (0.1)	0.6 (0.1)	1.2 (0.3)	3.3 (2.3)	1.2 (1.3)
Sociodemographic characteristics						
Sex, No. (%)						
Female	72 465 (83.6)	72 605 (78.1)	75 760 (68.0)	57 929 (49.3)	24 352 (23.4)	302 041 (58.9)
Male	14 215 (16.4)	20 324 (21.9)	35 633 (32.0)	59 625 (50.7)	79 793 (76.6)	210 660 (41.1)
Age, mean (SD), y	53.7 (13.3)	52.7 (12.1)	52.5 (11.1)	52.4 (10.9)	51.1 (13.4)	52.0 (10.7)
Urban residence, No. (%)	36 396 (42.0)	47 253 (50.8)	57 931 (52.0)	49 307 (41.9)	29 442 (28.3)	226 942 (44.3)
≥6 y Education, No. (%)	35 047 (40.4)	43 938 (47.3)	59 486 (53.4)	61 828 (52.6)	51 909 (49.8)	250 614 (48.9)
Household income ≥20 000 yuan, No. (%)	42 596 (49.1)	44 535 (47.9)	51 093 (45.9)	47 371 (40.3)	31 102 (29.9)	220 169 (42.9)
Environment						
Solid fuel for cooking,^b^ No. (%)						
Urban	5170 (14.4)	3815 (8.2)	2828 (4.9)	2246 (4.6)	1742 (4.8)	16 772 (7.4)
Rural	27 498 (54.2)	26 551 (57.4)	32 030 (59.9)	44 075 (64.5)	45 873 (67.8)	171 063 (59.7)
Season at time of survey,^c^ No. (%)						
Spring	25 064 (28.9)	27 475 (29.6)	31 976 (28.7)	35 164 (29.9)	33 922 (32.6)	153 749 (30.0)
Summer	17 242 (19.9)	23 668 (25.5)	31 681 (28.4)	30 472 (25.9)	18 221 (17.5)	125 732 (24.5)
Autumn	24 443 (28.2)	25 360 (27.3)	28 724 (25.8)	29 016 (24.7)	20 895 (20.1)	130 078 (25.4)
Winter	19 931 (23.0)	16 427 (17.7)	19 011 (17.1)	22 902 (19.5)	31 107 (29.9)	103 142 (20.1)
Lifestyle						
Physical activity, mean (SD), MET h/d	22.8 (17.3)	21.7 (15.0)	20.4 (13.0)	20.0 (12.4)	18.7 (15.2)	21.0 (13.0)
Ever regular alcohol drinking, No. (%)						
Male	5043 (35.0)	6936 (34.4)	12 798 (35.9)	22 014 (36.8)	30 780 (38.4)	78 049 (37.1)
Female	1542 (2.1)	1705 (2.3)	2004 (2.6)	1657 (2.9)	729 (3.0)	7646 (2.5)
Ever regular smoking, No. (%)						
Male	6850 (47.6)	10 003 (49.6)	19 068 (53.5)	44 721 (74.8)	74 964 (93.5)	156 650 (74.5)
Female	1083 (1.5)	1216 (1.7)	1707 (2.3)	2961 (5.1)	2967 (12.4)	9944 (3.3)
Physical measurements						
BMI, mean (SD)	23.4 (3.9)	23.8 (3.6)	24.0 (3.5)	23.8 (3.5)	23.7 (4.7)	23.7 (3.4)
SBP, mean (SD), mm Hg	131.9 (23.8)	131.5 (21.5)	130.7 (20.0)	131.2 (20.8)	133.0 (28.2)	131.3 (20.2)
RPG, mean (SD), mmol/L	5.9 (2.5)	6.1 (2.6)	6.2 (2.5)	6.1 (2.5)	6.0 (3.3)	6.1 (2.4)
FEV1/FVC, mean (SD), %	84.3 (11.0)	84.6 (9.7)	85.2 (8.5)	84.2 (7.8)	82.9 (9.4)	84.5 (8.4)
Health and medical history, No. (%)						
Poor self-reported health	9085 (10.5)	9873 (10.6)	11 168 (10.0)	12 744 (10.8)	12 839 (12.3)	53 387 (10.4)
COPD^d^	7687 (8.9)	7389 (8.0)	7668 (6.9)	7959 (6.8)	7212 (6.9)	37 483 (7.3)
Hypertension^d^	30 799 (35.5)	32 772 (35.3)	38 186 (34.3)	40 599 (34.5)	37 691 (36.2)	176 960 (34.5)
Diabetes^d^	4483 (5.2)	5525 (5.9)	7590 (6.8)	7604 (6.5)	6088 (5.8)	30 799 (6.0)
IHD	1490 (1.7)	2571 (2.8)	4197 (3.8)	4469 (3.8)	3421 (3.3)	15 743 (3.1)
Stroke/TIA	1059 (1.2)	1463 (1.6)	2092 (1.9)	2540 (2.2)	2255 (2.2)	9049 (1.8)

Abbreviations: BMI, body mass index (calculated as weight in kilograms divided by height in meters squared); CO, carbon monoxide; COHb, carboxyhemoglobin; COPD, chronic obstructive pulmonary disease; FEV1, forced expiratory volume in 1 second; FVC, forced vital capacity; IHD, ischemic heart disease; MET, metabolic equivalent of task; RPG, random plasma glucose; SBP, systolic blood pressure; SD, standard deviation; TIA, transient ischemic attack.

^a^ Data are standardized for age and sex, as appropriate, when these variables are not the variables of interest.

^b^ Solid fuel includes coal or wood.

^c^ Spring is defined as March-May; summer, June-August; autumn, September-November, winter, December-February.

^d^ Self-reported or screen-detected.

### Smoking and Exhaled CO Levels

**Figure 1.**
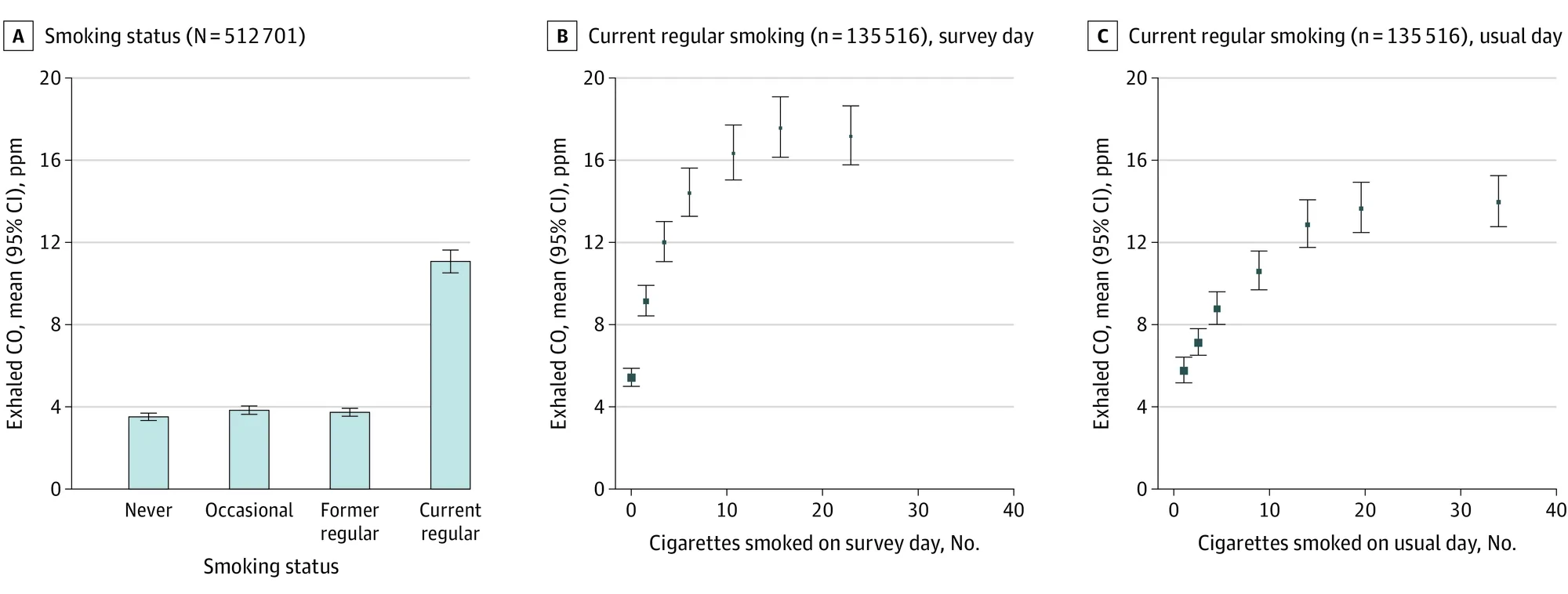
Bar Chart and Shape Plots Showing Exhaled Carbon Monoxide (CO) Levels by Smoking Status and Number of Cigarettes (or Equivalents) Smoked Per Day Geometric mean exhaled CO levels adjusted for age, sex, study area, solid fuel use, exposure to smoking (for those who do not smoke), and season and time of day when the survey was taken. The size of the squares in panels B and C is inversely proportional to the variance of the mean exhaled CO. The error bars indicate 95% CIs.

Overall, the mean exhaled CO levels were higher among those who smoked regularly (11.1 ppm; 95% CI, 10.5-11.7) than among those who never smoked (3.5 ppm; 95% CI, 3.4-3.7), those who occasionally smoked (3.8 ppm; 95% CI, 3.6-4.0), and those who formerly smoked regularly (3.7 ppm; 95% CI, 3.6-3.9) (Figure 1). Across all smoking categories, mean exhaled CO levels were higher in male individuals than female individuals (eFigure 3 in Supplement 1) and in rural than in urban areas (eFigure 4 in Supplement 1). Among those who regularly smoked at baseline, mean exhaled CO levels increased both with the number of cigarettes smoked on the survey day and on a usual day, which plateaued at approximately 12 to 14 and 15 to 20 cigarettes per day, respectively (Figure 1). These associations differed little by sex or by urban vs rural residence (eFigures 3-4 in Supplement 1).

### Incidence of PD

**Figure 2.**
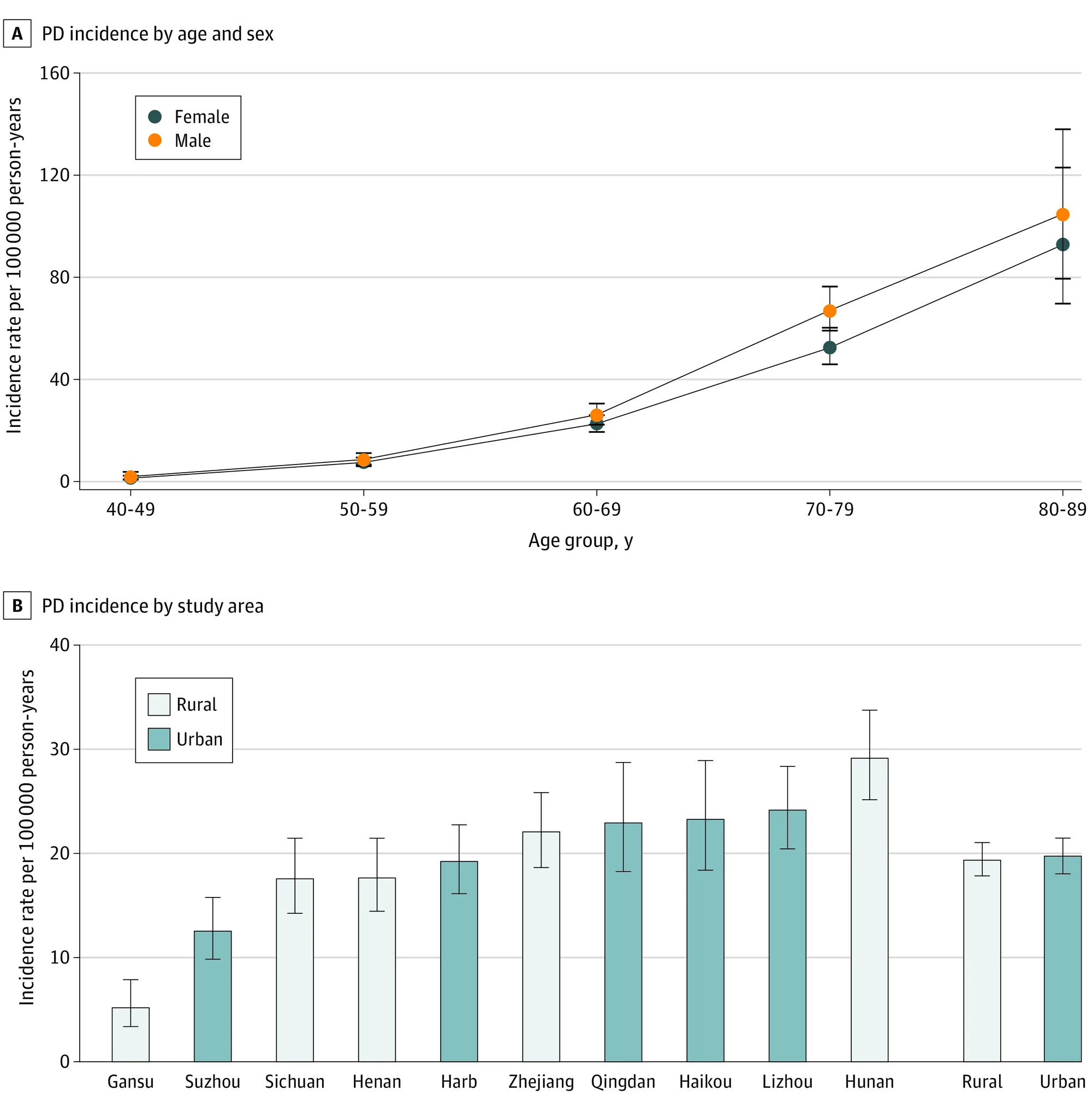
Line Graphs and Bar Charts Showing Incidence Rates of Parkinson Disease (PD) by Age, Sex, and Study Area Incidence rates for the full CKB cohort (N = 512 724) standardized by age, sex, and study area, as appropriate. Incidence rates were slightly higher in male individuals than in female individuals (22.1 per 100 000 person-years; 95% CI, 20.3-24.1 vs 17.7 per 100 000 person-years; 95% CI, 16.3-19.3, respectively) but similar in urban and rural areas (19.7; 95% CI, 18.0-21.4 vs 19.3; 95% CI, 17.8-21.0). Error bars represent 95% CIs.

During approximately 6 million person-years of follow-up (median duration per person: 12.1 years), 1131 cases of PD were recorded, with incidence rates increasing exponentially with age and slightly higher in male individuals than in female individuals (22.1 vs 17.7 per 100 000 person-years) (Figure 2) but similar in urban and rural areas (19.7; 95% CI, 18.0-21.4 vs 19.3; 95% CI, 17.8-21.0) (eFigure 5 in Supplement 1).

Among participants with PD, approximately 90% (n = 1012) had no recorded diagnosis of any other neurodegenerative disease during follow-up. A small proportion had additional neurodegenerative disease diagnoses recorded, including dementia (n = 82 [7.3%]), brain atrophy (n = 38 [3.4%]), and age-related macular degeneration (n = 5 [0.4%]) (eFigure 6 in Supplement 1). Additionally, 86 participants without a PD diagnosis had a recorded diagnosis of parkinsonism.

### Smoking, Exhaled CO, and Risk of PD and Other Diseases

**Figure 3.**
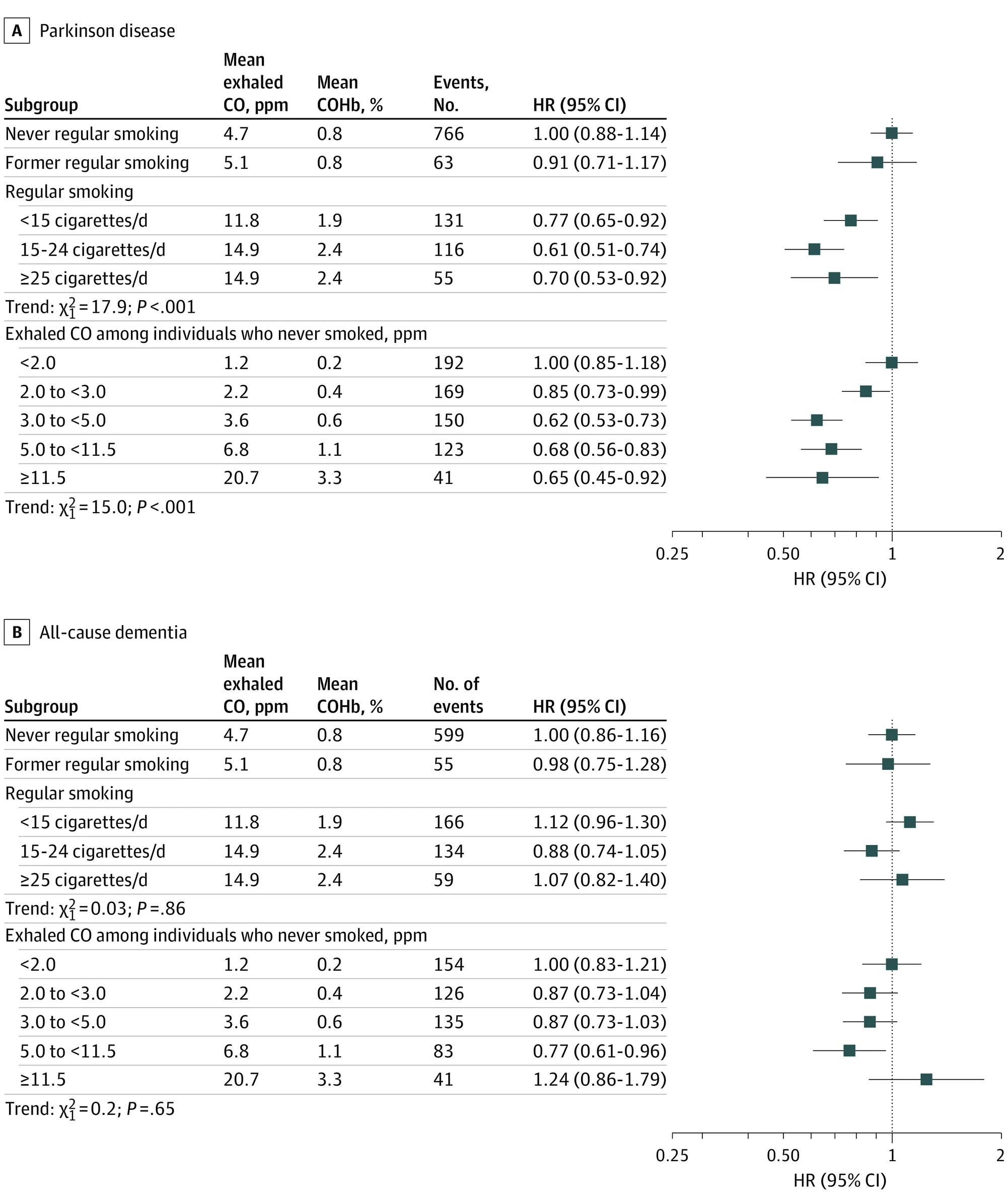
Forest Plots Showing Associations of Smoking and Exhaled Carbon Monoxide (CO) With Risks of Parkinson Disease and Dementia Hazard ratios (HRs) are stratified by age at risk (5-year groups), sex, and study area and adjusted for education, body mass index, alcohol consumption, and physical activity. Associations with exhaled CO were additionally adjusted for season and time of day at survey, solid fuel use, and passive exposure to smoking. The χ^2^ and *P* values assess evidence of a linear gradient across ordered strata. Cigarettes/d refers to cigarettes or equivalent products. COHb indicates carboxyhemoglobin.

Compared with those who never smoked regularly, those who smoked regularly had an approximately 30% lower risk of PD (HR, 0.70; 95% CI, 0.62-0.79) (eFigure 7 in Supplement 1). The risk of PD was slightly lower, albeit nonsignificant, among those who formerly smoked regularly (HR, 0.91; 95% CI, 0.71-1.17) and, among those who smoked regularly, the risk of PD decreased with increasing smoking intensity until around 15 to 24 cigarettes per day (<15/d: 0.77; 95% CI, 0.65-0.92; 15-24/d: 0.61; 95% CI, 0.51-0.74; ≥25/d: 0.70; 95% CI, 0.53-0.92; *P* for trend < .001) (Figure 3). There was a nonsignificant dose-response association by smoking duration, as measured in smoking pack-years; however, the risk did not vary by level of inhalation or by age at smoking initiation (eFigure 7 in Supplement 1). There was a slightly lower risk among those who smoked regularly after excluding those who stopped due to ill health (HR, 0.64; 95% CI, 0.55-0.73). Among individuals who never smoked, passive exposure to smoking was not clearly associated with risk of PD (eFigure 7 in Supplement 1).

Among individuals who never smoked, exhaled CO level was inversely associated with risk of PD in a dose-dependent manner. After further adjustment for factors related to exhaled CO (ie, season and hour at survey, solid fuel use, and passive exposure to smoking), the HRs were 1.00 (95% CI, 0.85-1.18) at exhaled CO <2.0 ppm, 0.85 (95% CI, 0.73-0.99) at 2.0 to <3.0 ppm, 0.62 (95% CI, 0.53-0.73) at 3.0 to <5.0 ppm, 0.68 (95% CI, 0.56-0.83) at 5.0 to <11.5 ppm, and 0.65 (95% CI, 0.45-0.92) at ≥11.5 ppm (*P* for trend < .001) (Figure 3). Similar associations were found in individuals who never smoked and in those who smoked regularly using group-specific exhaled CO quintiles of exposure (eFigure 8 in Supplement 1), after additionally excluding participants with major chronic diseases at baseline (eFigure 9 in Supplement 1), or from Henan (eFigure 10 in Supplement 1), in models adjusting for different covariates (eTable 5 in Supplement 1) or when extending the outcome to all-cause parkinsonism (eFigure 11 in Supplement 1). Among individuals who never smoked, exhaled CO levels ≥3 ppm were associated with an approximately 30% lower risk of PD (HR, 0.71; 95% CI, 0.59-0.84) compared to levels <3 ppm (eFigure 12 in Supplement 1). These associations differed little by individual study area (eFigure 13 in Supplement 1) and across major population subgroups (eFigures 12 and 14 in Supplement 1). However, a dose-response association of exhaled CO with PD was present among female individuals who never smoked (HR, 1.00; 95% CI, 0.84-1.19; HR, 0.84; 95% CI, 0.71-0.99; HR, 0.62; 95% CI, 0.51-0.74; HR, 0.63; 95% CI, 0.51-0.79; and HR, 0.57; 95% CI, 0.38-0.85 for exhaled CO at <2.0, 2.0 to <3.0, 3.0 to <5.0, 5.0 to <11.5, and ≥11.5 ppm, respectively; *P* for trend < .001) and in rural areas where a high proportion of individuals who never smoked (90%) were female (eFigure 14 in Supplement 1). The association did not vary materially when using different definitions of never smoking such as fewer than 100 cigarettes or equivalent per lifetime (eFigure 15 in Supplement 1) or when restricting the PD case definition to participants with more than 1 recorded diagnosis of PD during follow-up (n = 597 events; HR, 0.67; 95% CI, 0.53-0.85 comparing exhaled CO levels of ≥3 ppm vs <3 ppm among individuals who never smoked).

Ever-regular smoking was associated with higher risk of macular degeneration in male individuals (HR, 1.45; 95% CI, 1.03-2.03), but there was otherwise no clear dose-dependent association of smoking or exhaled CO with risks of other neurodegenerative diseases (Figure 3; eFigures 16 and 17 in Supplement 1). When allowing for coexisting diagnoses of other neurodegenerative diseases and parkinsonism, there was a weak inverse association of exhaled CO (but not smoking) with dementia, which disappeared at levels ≥11.5 ppm (eFigures 18 and 19 in Supplement 1).

**Figure 4.**
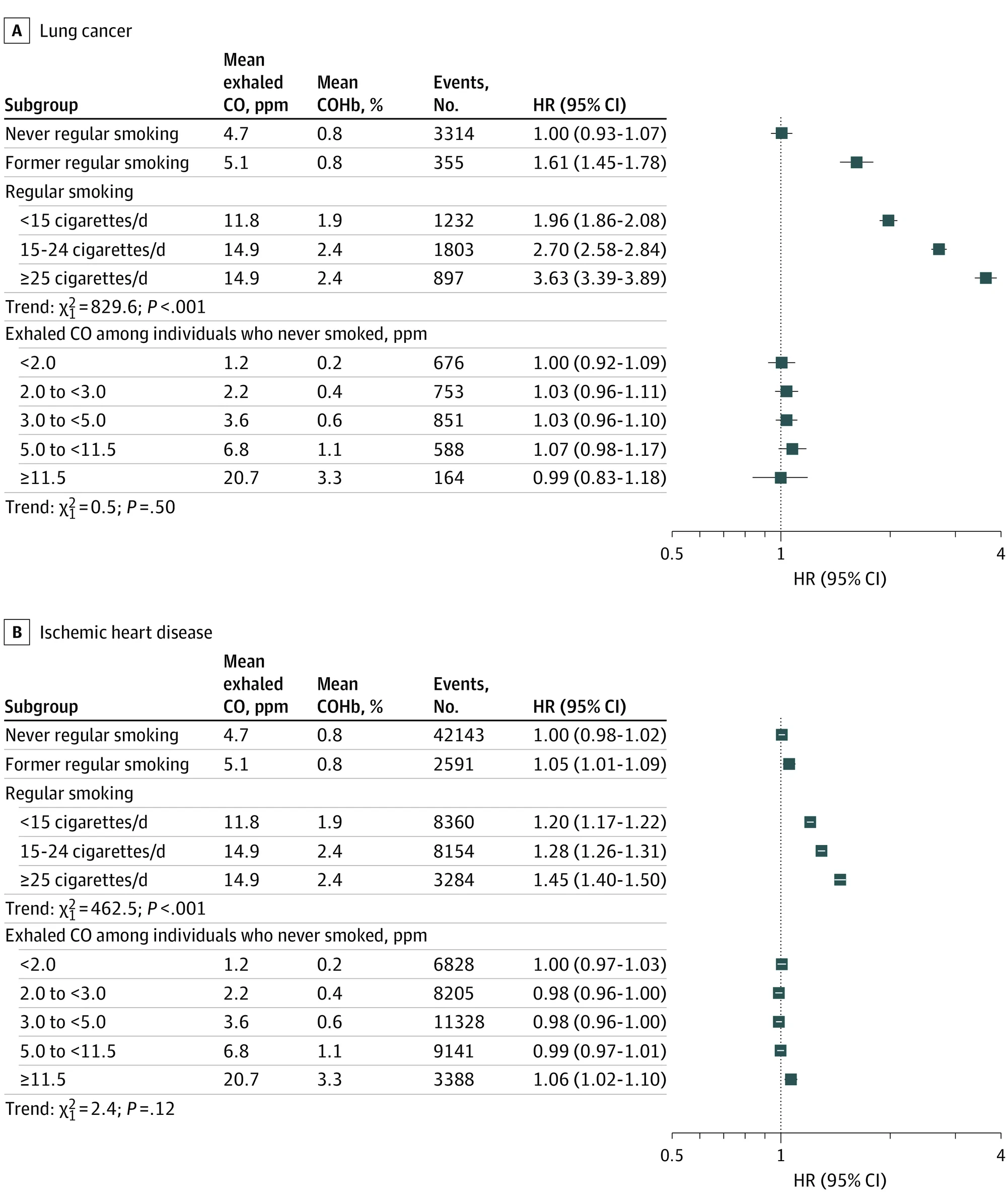
Forest Plots Showing Associations of Smoking and Exhaled Carbon Monoxide (CO) With Risks of Lung Cancer and Ischemic Heart Disease Hazard ratios (HRs) are stratified by age at risk (5-year groups), sex, and study area and adjusted for education, body mass index, alcohol consumption, and physical activity. Associations with exhaled CO were additionally adjusted for season and time of day at survey, solid fuel use, and passive exposure to smoking. The χ^2^ and *P* values assess evidence of a linear gradient across ordered strata. Cigarettes/d refers to cigarettes or equivalent products. COHb indicates carboxyhemoglobin.

There were positive, dose-dependent associations between smoking and risks of major smoking-related diseases, including lung cancer, ischemic heart disease, stroke, and all-cause mortality (Figure 4; eFigure 20 in Supplement 1). Among individuals who never smoked, however, there were no associations of exhaled CO with risks of any of these smoking-related outcomes.

## Discussion

This cohort study provides epidemiological evidence supporting the role of CO in PD etiology. In addition to supporting the inverse association between smoking and PD in Chinese adults, our study demonstrates the potential role of CO in mediating such associations in epidemiological studies. These findings help to refute the presumed protective effects on PD of tobacco smoking specifically and reveal a novel etiologic clue relevant for development of new PD prevention and treatment measures.

In China, the burden of PD has consistently risen in the last decades, partly due to population aging. In 2021, the age-standardized PD incidence rate was estimated at 24.3 per 100 000 population, much higher than the global average (15.6).^40^ Although not designed to be nationally representative, the incidence rate of PD in CKB (19.5 per 100 000 person-years) was similar to estimates for China and the Western Pacific region.^41^ Moreover, as in previous research, we found higher rates in male individuals than female individuals. However, the sex difference in incidence rates (22.1 vs 17.7, equivalent to a male to female ratio of 1.25) was somewhat smaller than that reported in European and US populations (approximately 1.5), although generally consistent with the lower male to female ratios reported in many Asian populations.^42,43^ This may partly reflect the much higher smoking prevalence among male individuals in CKB (74.5% vs 3.3%) than in European and US populations, consistent with reported international patterns linking smoking with sex differences in PD.^44^ We measured exhaled CO among all study participants to objectively validate self-reported smoking and other environmental sources of CO exposure. Indeed, the exhaled CO levels were approximately 3-fold higher in those who regularly smoked than in those who previously smoked regularly, those who occasionally smoked, and those who never smoked. Moreover, among those who smoked regularly, exhaled CO levels increased progressively with smoking intensity. The measurement of exhaled CO among all participants in CKB not only helped to confirm the validity of self-reported smoking exposures but also, and more importantly, provided a unique opportunity to assess the likely independent role of CO as a plausible mechanism underlying the smoking-PD association.^4,22^

As in previous observational studies, we found an inverse association between smoking and risk of PD in Chinese adults. However, the risk estimate comparing those who regularly vs never regularly smoked was somewhat weaker than that reported in studies of European and US populations, which generally showed a halving of the risk.^3,8,9,10^ The smaller risk estimate in Chinese adults may be attributed to less prolonged persistent cigarette smoking among adults in China compared with European and US populations.^45^ Nevertheless, our study, with detailed smoking assessment and exhaled CO measurements, provides evidence supporting an association of exhaled CO (uncoupled from smoking) with PD in humans. The dose-dependent inverse association of exhaled CO with PD risk observed among individuals who never smoked suggests a possible new mechanism underlying the smoking-PD association that is not explained by nicotine addiction or other cigarette components. Notably, the association was evident at relatively low exhaled CO levels among individuals who never smoked, with signs of plateauing at around 5 ppm, a level well below the mean exhaled CO level in those who regularly smoked (approximately 11 ppm). The association between exhaled CO and PD was robust after controlling for environmental factors, including solid fuel use and passive exposure to smoking, and was generally consistent across population subgroups. Similarly, the association was unchanged after excluding participants with prevalent chronic disease, suggesting the findings are not confounded by unrelated comorbidities. The observed association was specific to PD, with no association found between exhaled CO and risk of lung cancer, ischemic heart disease, stroke, or overall mortality, nor with other neurodegenerative diseases. In sex-specific analyses, we found a much clearer and somewhat stronger dose-response association between exhaled CO and PD in female individuals. While this may represent a statistical artifact due to a relatively smaller number of PD cases among male individuals who never smoked, the amplification of the association in female individuals could also be due to established sex differences in hemoglobin metabolism and endogenous CO production over the life course.^22^

While the half-life of CO in the body following acute exposure is only a few hours,^46^ we found an association with incident PD after many years of follow-up, suggesting exhaled CO measured once at baseline may at least crudely reflect long-term exogenous and endogenous levels of CO exposure.^34^ In the absence of smoking and major environmental exposures, the amount of exogenous CO is often relatively small compared with endogenous body stores.^47^ Low levels of CO are produced continuously as a byproduct of heme degradation and CO is involved in regulating many physiological processes throughout the body.^22^ CO binds to hemoglobin with greater affinity than oxygen, reducing its oxygen-carrying capacity. This property, while contributing to CO’s toxicity, is also believed to underlie most of its pharmacological functions.^47^ Heme oxygenase 1, the primary inducible stress isozyme that generates CO, has a role in protecting against oxidative damage and inflammation.^20,21,22^ Heme oxygenase 1 is highly upregulated by nuclear factor erythroid 2-related factor 2 (Nrf2), which has been found to be associated with PD,^4,48,49^ in response to a vast number of pro-oxidant stimuli, including smoking and the antioxidant response initiated by CO itself, allowing positive feedback over the system.^21,50,51^ Heme oxygenase 1 expression has been independently associated with PD and Lewy bodies,^4,52,53^ with some, albeit limited, evidence on heme oxygenase genetic polymorphisms associated with PD.^4^ Collectively, the heme oxygenases are closely involved with hypoxia and oxygen-dependent processes.^23^ Hypoxic conditioning in PD has gained interest in recent years, with preclinical evidence suggesting neuroprotective effects and an early N-of-1 trial suggesting specific protocols are not only safe and well tolerated in patients with PD, but may also be associated with short-term symptom improvement.^54,55^ While existing evidence linking CO and brain health is still in its infancy, some research suggested a broad neuroprotective effect of CO,^26,56,57^ which, if true, could be relevant for a range of neurological diseases. With the exception of PD, however, we did not find any clear associations of exhaled CO with risks of other neurodegenerative diseases. Nevertheless, our findings suggested a biologically plausible mechanism involving CO, which, if more broadly neuroprotective, could mask, to a certain extent, the deleterious effects of smoking on brain health in epidemiological studies.

Apart from the prospective design, large sample size, and ability to study a wide range of fatal and nonfatal health outcomes, the key strength of our study is the measurement of exhaled CO among all 0.5 million participants, making it the first large prospective study, to our knowledge, to explore the associations of exhaled CO with PD in humans. Moreover, since smoking is largely limited to male individuals in China,^45,58^ we were able to reliably examine the association of exhaled CO with PD independent of smoking in a large, mostly female, never-smoking population, minimizing bias from misclassification of smoking exposure. Additionally, the depth of information gathered at the time of survey, which included passive exposure to smoking and environmental factors, such as solid fuel use, allowed estimates to be adjusted for these factors and for the consistency of the association to be investigated and established across different population subgroups. Furthermore, our analyses included multiple smoking-related diseases (eg, lung cancer, ischemic heart disease, stroke, and overall mortality) and other neurodegenerative conditions, which helped to establish the specificity of the exhaled CO–PD association.

### Limitations

This study has limitations. First, the relatively young mean age of community-based participants without disability at recruitment and limited follow-up data from primary care or institutionalized settings may have contributed to relatively low overall PD incidence rates in CKB. This has implications for generalizability of the study findings given these and other differences between populations; for example, in characteristic smoking patterns, exposure to solid fuel use and ambient air pollution, and in health care systems. Moreover, this resulted in reduced statistical power, particularly in male individuals among whom most smoked, to further explore sex-specific associations of exhaled CO and PD and the shape of the dose-response association among individuals who never smoked. The relatively low incidence of PD may also reflect misdiagnosis or underdiagnosis. However, in ongoing adjudication of neurological diseases in CKB, 94% of PD cases reviewed to date have been validated, demonstrating overall robust characterization of the disease. Second, a single baseline measurement of exhaled CO is a potentially crude measurement of long-term CO exposure. In a subset of about 20 000 participants who participated in a resurvey approximately 2.5 years after the baseline survey, the overall correlation of measured exhaled CO between baseline and resurvey was approximately 0.6, likely reflecting, at least in part, recognized changes over time^59,60^ in smoking behaviors, type of cooking and heating fuels used, frequency of cooking, ventilation, and seasons of measurements between 2 time points. Importantly, however, resulting intraindividual variation in exhaled CO levels would lead to regression dilution bias^61,62^ and consequent underestimation of the true association of exhaled CO levels with risk of PD in the present study. Although adjustment was made for major environmental sources of CO, residual confounding from ambient air pollution and changes in solid fuel use over time^60^ cannot be fully excluded. However, such confounding by change in environmental exposures would be expected to bias associations toward the null rather than generate spurious inverse associations. Furthermore, sensitivity analyses showed no indication that the exhaled CO–PD association was driven by any single study area. Third, we were unable to exclude the possibility that nicotine or other tobacco components also contributed to the association between smoking and PD. Fourth, although attempts were made to adjust for environmental sources of CO exposure, we were unable to fully account for these and to reliably distinguish between endogenous and exogenous CO, which may inform assessment of causality and understanding of the biological pathways underlying the association. Although the findings were robust, more evidence from large prospective studies with measurement of exhaled CO is needed to replicate our findings in other populations.

## Conclusions

In summary, this cohort study demonstrated a clear dose-dependent inverse association between exhaled CO and risk of PD in individuals who never smoked, suggesting CO may play a role in mediating the inverse association between smoking and PD. Our findings help to refute the presumed protective effects on PD associated with tobacco smoking specifically, further strengthening the need for tobacco control. Clinical trials have indicated that low-dose CO can be administered safely within defined exposure ranges,^63^ and trials are under way to specifically assess the safety, tolerability, and pharmacokinetics of HBI-002, an oral low-dose CO liquid drug product, in patients with PD (NCT07005180). The present study provides evidence supporting the scientific rationale and potential for success of such an intervention for PD. If successful, this new treatment could contribute to reducing the global burden of PD in the future.

## Notes

Supplement 1. eTable 1. Typical values of exhaled CO and carboxyhaemoglobin by smoking status eTable 2. Definition of disease outcomes and number of first events eTable 3. Baseline characteristics of study participants by incident Parkinson disease diagnosis eTable 4. Baseline characteristics of never smokers by exhaled CO level in males and females eTable 5. Association of exhaled CO with risk of Parkinson disease among never smokers by degree of adjustment eFigure 1. Definitions of smoking exposure and number of participants eFigure 2. Proportion of participants with different levels of exhaled CO among never smokers, by study area and sex eFigure 3. Exhaled CO levels by smoking status and number of cigarettes or equivalents smoked per day in males and females eFigure 4. Exhaled CO levels by smoking status and number of cigarettes or equivalents smoked per day, by urban and rural residence eFigure 5. Incidence rates of Parkinson disease by age and study area eFigure 6. Number of participants with diagnoses of different neurodegenerative diseases eFigure 7. Association of smoking with risk of Parkinson disease, by smoking characteristics eFigure 8. Association of exhaled CO with risk of Parkinson disease among never smokers and regular smokers, by group-specific quintiles eFigure 9. Associations of exhaled CO with risks of selected disease outcomes, applying sequential exclusion criteria eFigure 10. Association of exhaled CO with risk of Parkinson disease among never smokers, excluding participants from Henan eFigure 11. Association of smoking and exhaled CO with risk of parkinsonism eFigure 12. Adjusted HRs for risk of Parkinson disease associated with high exhaled CO (≥3 ppm vs <3 ppm) among never smokers, in various participant subgroups eFigure 13. Association of exhaled CO with risk of Parkinson disease among never smokers, by study area eFigure 14. Association of exhaled CO with risk of Parkinson disease among never smokers, by sex and urban or rural residence eFigure 15. Association of exhaled CO with risk of Parkinson disease by smoking characteristics, in males eFigure 16. Association of smoking and exhaled CO with risk of brain atrophy and macular degeneration eFigure 17. Association of smoking and exhaled CO with risk of dementia, by disease subtype eFigure 18. Association of smoking and exhaled CO with risks of other neurodegenerative diseases, without excluding co-diagnoses of parkinsonism eFigure 19. Association of smoking and exhaled CO with risks of dementia subtypes, without excluding co-diagnoses of parkinsonism eFigure 20. Association of smoking and exhaled CO with risks of stroke and all-cause mortality

Supplement 2. China Kadoorie Biobank Collaborative Group members

Supplement 3. Data sharing statement
