# Supplementary material for: Smoking, Exhaled Carbon Monoxide, and Risk of Parkinson Disease
Source: JAMA Neurol. 2026 Sep 8:e263037. Online ahead of print. doi: 10.1001/jamaneurol.2026.3037 (PMC13555506; doi:10.1001/jamaneurol.2026.3037)
Supplement: Supplementary file 1 [file jamaneurol-e263037-s001.pdf]

Supplemental Online Content

Bueno Lopez C, Iona A, Turnbull I, et al; China Kadoorie Biobank Collaborative Group. Smoking, exhaled carbon monoxide, and risk of Parkinson disease. *JAMA Neurol*. Published online September 8, 2026. doi:10.1001/jamaneurol.2026.3037

eTable 1. Typical values of exhaled CO and carboxyhaemoglobin by smoking status
eTable 2. Definition of disease outcomes and number of first events
eTable 3. Baseline characteristics of study participants by incident Parkinson disease diagnosis
eTable 4. Baseline characteristics of never smokers by exhaled CO level in males and females
eTable 5. Association of exhaled CO with risk of Parkinson disease among never smokers by degree of adjustment
eFigure 1. Definitions of smoking exposure and number of participants
eFigure 2. Proportion of participants with different levels of exhaled CO among never smokers, by study area and sex
eFigure 3. Exhaled CO levels by smoking status and number of cigarettes or equivalents smoked per day in males and females
eFigure 4. Exhaled CO levels by smoking status and number of cigarettes or equivalents smoked per day, by urban and rural residence
eFigure 5. Incidence rates of Parkinson disease by age and study area
eFigure 6. Number of participants with diagnoses of different neurodegenerative diseases
eFigure 7. Association of smoking with risk of Parkinson disease, by smoking characteristics
eFigure 8. Association of exhaled CO with risk of Parkinson disease among never smokers and regular smokers, by group-specific quintiles
eFigure 9. Associations of exhaled CO with risks of selected disease outcomes, applying sequential exclusion criteria
eFigure 10. Association of exhaled CO with risk of Parkinson disease among never smokers, excluding participants from Henan
eFigure 11. Association of smoking and exhaled CO with risk of parkinsonism
eFigure 12. Adjusted HRs for risk of Parkinson disease associated with high exhaled CO (≥3 ppm vs <3 ppm) among never smokers, in various participant subgroups
eFigure 13. Association of exhaled CO with risk of Parkinson disease among never smokers, by study area
eFigure 14. Association of exhaled CO with risk of Parkinson disease among never smokers, by sex and urban or rural residence
eFigure 15. Association of exhaled CO with risk of Parkinson disease by smoking characteristics, in males
eFigure 16. Association of smoking and exhaled CO with risk of brain atrophy and macular degeneration
eFigure 17. Association of smoking and exhaled CO with risk of dementia, by disease subtype
eFigure 18. Association of smoking and exhaled CO with risks of other neurodegenerative diseases, without excluding co-diagnoses of parkinsonism

© 2026 Bueno Lopez C et al. *JAMA Neurology*.

eFigure 19. Association of smoking and exhaled CO with risks of dementia subtypes, without excluding co-diagnoses of parkinsonism
eFigure 20. Association of smoking and exhaled CO with risks of stroke and all-cause mortality

This supplemental material has been provided by the authors to give readers additional information about their work.

© 2026 Bueno Lopez C et al. *JAMA Neurology*.

eTable 1: Typical values of exhaled CO and carboxyhaemoglobin by smoking status

COex, ppm	COHb (%)	Cigarette consumption
0 – 6	0 – 1	Non smoker
7 – 10	1.1 – 1.6	Light smoker
11 – 72	1.8 – 12	Heavy smoker
>72	> 12	Suspected poisoning

COHb=carboxy haemoglobin.
Source: CareFusion MicroCO Operating Manual.

© 2026 Bueno Lopez C et al. *JAMA Neurology*.

eTable 2: Definition of disease outcomes and number of first events

	No. of first events*	Definition and ICD-10 codes
**Parkinson's disease**	1,131	G20
**Parkinsonism**	1,217	G20; G21; G22; G23.0; G23.1; G23.2; G23.3; G23.8; G23.9; G25.9; G26; G90.3
**Dementia**		
Vascular	173	F01; I67.3
Alzheimer's	345	F00; G30
Other	648	F02; F03; F05.1; G31.0; G31.1; G31.8; A81.0; F10.6
All-cause	1,102	F01; I67.3; F00; G30; F02; F03; F05.1; G31.0; G31.1; G31.8; A81.0; F10.6
**Brain atrophy**†	1,471	G31.9
**AMD**	564	H35.3
**Lung cancer**	7,610	C33-C34
**IHD**	64,778	I20-I25
**Stroke**	69,821	I60-I61, I63-I64
**All-cause mortality**	56,545	Death due to any cause

* No. of first events shown are not mutually exclusive.

† Identified primarily via brain imaging.

Abbreviations: AMD=age-related macular degeneration; IHD=ischaemic heart disease.

© 2026 Bueno Lopez C et al. *JAMA Neurology*.

eTable 3: Baseline characteristics of study participants by incident Parkinson's disease diagnosis

	Parkinson's disease	
	**No** (n=511,570)	**Yes** (n=1,131)
**COex**	7.3 (7.2)	5.3 (6.7)
**Estimated COHb%**	1.2 (1.1)	0.8 (1.1)
**Socio-demographics**		
Female	58.9	51.7
Age, years	52.0 (10.6)	61.3 (9.8)
Urban residence	44.3	39.2
≥6 years of education	48.9	41.7
Household income ≥20,000 yuan	42.9	38.2
**Environment**		
Solid fuel for cooking*		
Urban	7.4	5.5
Rural	59.7	54.0
Season at survey†		
Spring	30.0	28.7
Summer	24.5	19.8
Autumn	25.4	22.6
Winter	20.1	16.7
**Lifestyle**		
Physical activity, MET-h/day	21.0 (11.8)	16.9 (14.9)
Ever regular alcohol drinking		
Males	37.1	29.9
Females	2.5	1.6
Ever regular smoking		
Males	74.5	57.1
Females	3.3	2.3
**Physical measurements**		
BMI, kg/m²	23.7 (3.2)	21.0 (3.5)
SBP, mmHg	131.3 (19.8)	115.8 (20.4)
RPG, mmol/L	6.1 (2.3)	5.3 (2.1)
FEV1/FVC, %	84.5 (7.6)	74.4 (6.9)
**Health and medical history**		
Poor self-reported health	10.4	14.6
COPD‡	7.3	4.8
Hypertension‡	34.5	32.0
Diabetes‡	6.0	5.7
IHD	3.1	3.2
Stroke/TIA	1.8	3.5

Data presented are mean (SD) or %. Standardized for age, sex and region, as appropriate.
* Solid fuel=coal or wood. †Spring= March-May; Summer= June-August; Autumn= September-November, Winter= December-February.
‡ Self-reported or screen-detected.
Abbreviations: COHb=carboxy haemoglobin. MET=metabolic equivalent of task; BMI=body mass index; SBP=systolic blood pressure; RPG=random plasma glucose; FEV1=forced expiratory volume in 1 second; FVC=forced vital capacity; COPD=chronic obstructive pulmonary disease; IHD=ischaemic heart disease; TIA=transient ischaemic attack.

© 2026 Bueno Lopez C et al. *JAMA Neurology*.

eTable 4: Baseline characteristics of never-smokers by exhaled CO level in males and females

	Males						Females					
	COex, ppm					Total	COex, ppm					Total
	**<2.0** (n=4,537)	**2.0 to <3.0** (n=5,849)	**3.0 to <5.0** (n=9,899)	**5.0 to <11.5** (n=7,848)	**≥11.5** (n=2,169)	(n=30,302)	**<2.0** (n=69,901)	**2.0 to <3.0** (n=70,472)	**3.0 to <5.0** (n=72,641)	**5.0 to <11.5** (n=53,561)	**≥11.5** (n=20,600)	(n=287,175)
**COex**	1.3 (0.3)	2.2 (0.3)	3.6 (0.5)	6.7 (1.7)	19.0 (10.1)	4.9 (5.2)	1.2 (0.3)	2.2 (0.2)	3.6 (0.5)	6.9 (1.8)	20.9 (11.5)	4.5 (5.9)
**Estimated COHb%**	0.2 (0.0)	0.4 (0.0)	0.6 (0.1)	1.1 (0.3)	3.0 (1.6)	0.8 (0.8)	0.2 (0.0)	0.4 (0.0)	0.6 (0.1)	1.1 (0.3)	3.4 (1.9)	0.7 (1.0)
**Socio-demographics**												
Age, years	56.0 (11.6)	55.3 (11.7)	54.0 (11.9)	53.6 (12.2)	53.3 (12.0)	54.4 (11.9)	51.8 (10.7)	50.8 (10.3)	51.0 (10.3)	51.0 (10.3)	50.8 (10.0)	51.1 (10.4)
Urban residence	54.4	58.0	61.8	52.6	34.9	55.6	41.9	50.9	53.1	42.0	16.2	45.1
≥6 years of education	59.1	62.9	68.8	67.8	65.5	65.7	33.5	41.4	49.4	51.4	44.6	43.6
Household income ≥20,000 yuan	52.6	50.6	50.8	45.0	35.2	48.5	46.6	45.9	43.4	36.3	22.7	41.9
**Environment**												
Solid fuel for cooking*	17.5	15.6	12.0	14.7	23.2	15.1	48.9	42.6	41.7	55.1	81.6	49.1
Season at survey†												
Spring	28.1	30.3	30.0	30.3	39.2	30.5	30.7	29.9	29.0	31.3	33.8	30.4
Summer	20.3	25.5	27.4	25.1	12.0	24.2	21.2	26.2	29.3	26.5	11.2	24.7
Autumn	27.7	27.8	25.3	23.4	15.3	25.0	28.2	27.3	25.7	24.1	17.1	25.8
Winter	23.9	16.4	17.3	21.1	33.5	20.3	19.9	16.7	16.0	18.1	37.8	19.1
Passive smoking exposure‡	21.4	23.7	22.6	24.6	24.1	23.3	36.6	40.3	43.1	46.4	47.0	41.7
**Physical measurements**												
BMI, kg/m²	23.4 (3.1)	23.6 (3.2)	24.1 (3.3)	24.1 (3.3)	24.0 (3.3)	23.9 (3.3)	23.4 (3.3)	23.8 (3.4)	24.1 (3.5)	24.1 (3.5)	24.4 (3.6)	23.8 (3.5)
SBP, mmHg	133.2 (19.4)	133.5 (19.0)	133.7 (19.6)	134.2 (19.6)	136.3 (19.8)	133.9 (19.4)	130.4 (20.4)	129.9 (20.4)	128.9 (20.3)	130.0 (20.8)	134.8 (21.2)	130.2 (20.6)
RPG, mmol/L	5.9 (2.1)	6.1 (2.4)	6.2 (2.5)	6.2 (2.6)	5.9 (2.4)	6.1 (2.4)	6.0 (2.0)	6.1 (2.3)	6.3 (2.4)	6.2 (2.5)	6.1 (2.5)	6.1 (2.3)
FEV1/FVC, %	85.4 (9.1)	85.3 (8.7)	86.2 (8.6)	85.4 (8.5)	83.1 (8.9)	85.5 (8.7)	85.1 (8.3)	85.3 (8.4)	85.8 (7.9)	84.4 (7.0)	82.6 (6.3)	85.0 (7.9)
**Health and medical history**												
Poor self-reported health	8.4	9.0	7.4	8.0	8.8	8.1	10.2	10.4	10.4	12.0	14.5	11.0
COPD§	8.7	8.4	6.8	6.7	8.3	7.5	6.7	6.0	5.3	5.1	4.9	5.7
Hypertension§	38.6	39.4	39.6	39.4	41.4	39.5	32.8	32.6	31.5	33.0	39.3	32.9
Diabetes§	5.9	6.6	7.4	8.1	6.1	7.1	5.0	5.9	6.9	6.8	6.3	6.1
IHD	2.5	3.3	4.4	4.5	4.2	3.9	1.7	2.9	4.1	4.4	3.3	3.2
Stroke/TIA	2.1	2.8	2.9	3.5	3.3	2.9	0.8	1.1	1.3	1.9	2.0	1.3

Data presented are mean (SD) or %. Standardized for age, as appropriate.
*Solid fuel= coal or wood. †Spring= March-May; Summer= June-August; Autumn= September-November, Winter= December-February. ‡Daily or almost every day. §Self-reported or screen-detected.
Abbreviations: COHb=carboxy haemoglobin. FEV1=forced expiratory volume in 1 second; FVC=forced vital capacity; COPD=chronic obstructive pulmonary disease; IHD=ischaemic heart disease; TIA=transient ischaemic attack.

© 2026 Bueno Lopez C et al. *JAMA Neurology*.

eTable 5: Association of exhaled CO with risk of Parkinson's disease among never smokers by degree of adjustment

COex, ppm	N event	Adjusted HR (95% CI)			
		Model 1	Model 2	Model 3	Model 4
<2.0	192	1.00 (0.85, 1.18)	1.00 (0.85, 1.18)	1.00 (0.85, 1.18)	1.00 (0.85, 1.18)
2.0 to <3.0	169	0.85 (0.73, 0.99)	0.85 (0.73, 0.99)	0.85 (0.73, 0.99)	0.85 (0.73, 0.99)
3.0 to <5.0	150	0.62 (0.53, 0.73)	0.62 (0.53, 0.73)	0.62 (0.53, 0.73)	0.62 (0.53, 0.73)
5.0 to <11.5	123	0.68 (0.56, 0.82)	0.68 (0.56, 0.82)	0.67 (0.56, 0.81)	0.68 (0.56, 0.83)
≥11.5	41	0.63 (0.44, 0.90)	0.63 (0.44, 0.90)	0.63 (0.44, 0.89)	0.65 (0.45, 0.92)
LRT* (χ^2, df, p)		(18.8, 4, <0.001)	(18.8, 4, <0.001)	(19.2, 4, <0.001)	(18.2, 4, =0.001)

*LRT=Likelihood ratio test comparing model fit before and after adding COex. Model 1: HRs are stratified by age-at-risk (5-year groups), sex, and study area and adjusted for education. Model 2: Model 1, additionally adjusted for income. Model 3: Model 1, additionally adjusted for BMI, alcohol drinking and physical activity. Model 4: Model 3, additionally adjusted for season, time of day at survey, solid fuel use and exposure to passive smoking.

© 2026 Bueno Lopez C et al. *JAMA Neurology*.

eFigure 1: Definitions of smoking exposure and number of participants

Note: Former smokers had ceased smoking regularly for at least 6 months.

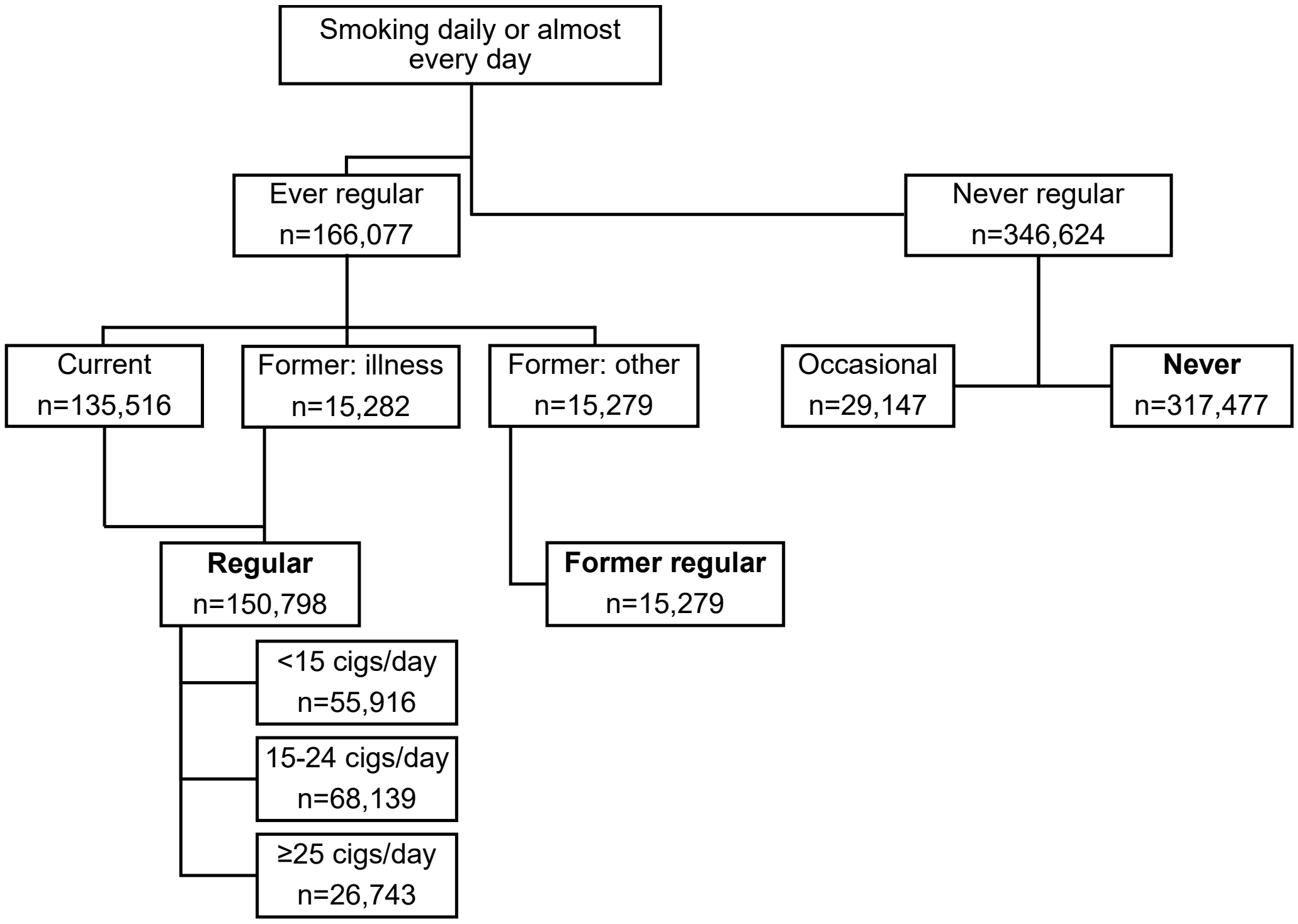

© 2026 Bueno Lopez C et al. *JAMA Neurology*.

eFigure 2: Proportion of participants with different levels of exhaled CO among never smokers, by study area and sex

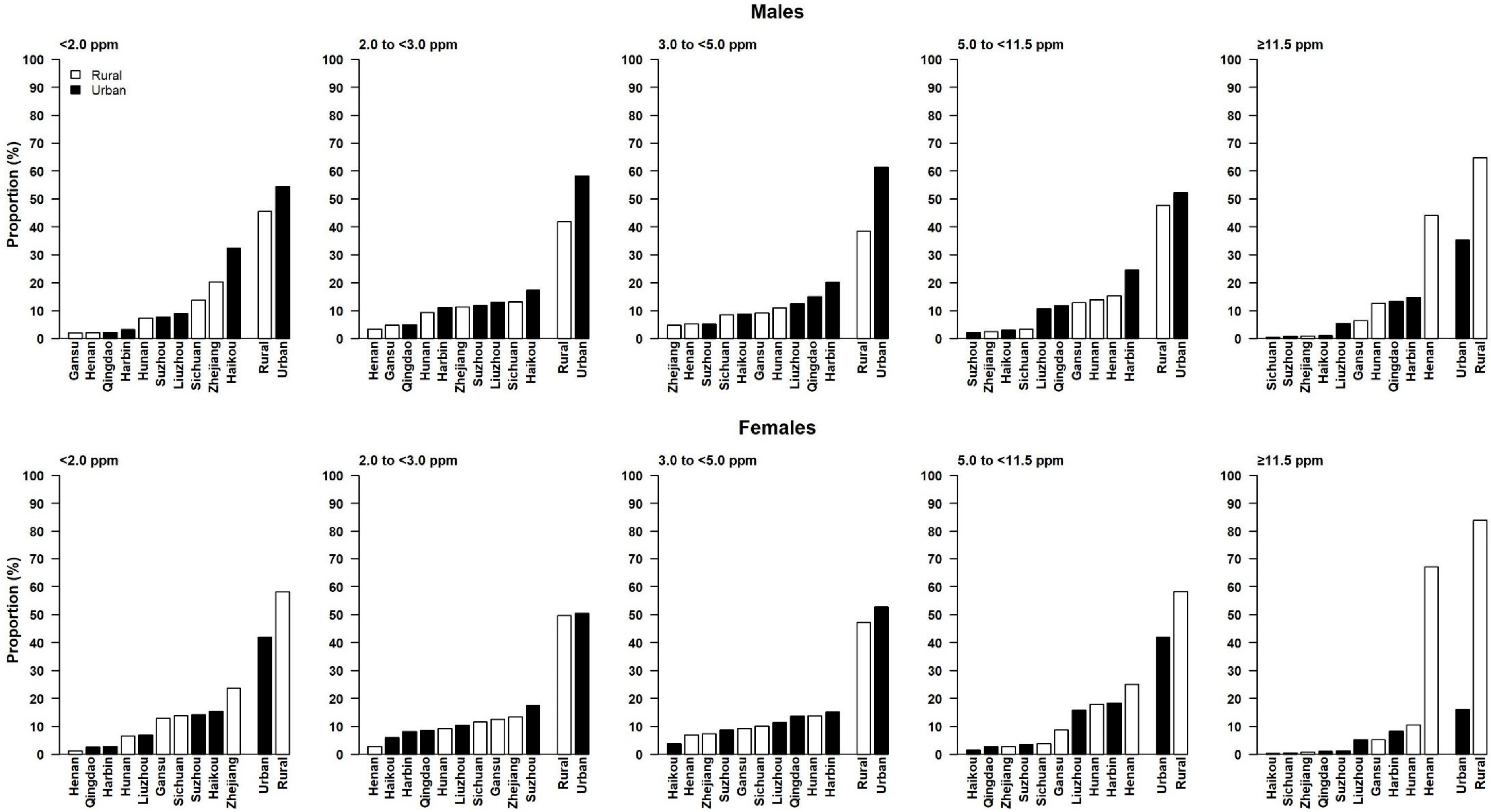

© 2026 Bueno Lopez C et al. *JAMA Neurology*.

eFigure 3: Exhaled CO levels by smoking status and number of cigarettes (or equivalents) smoked per day in males and females

Geometric mean COex levels adjusted for age, study area, solid fuel use, exposure to passive smoking (for non-smokers), season, and time of day at survey. The area of the squares is inversely proportional to the variance of the mean COex. Vertical lines indicate 95% CIs. Numbers above the 95% CI are point estimates for adjusted means.

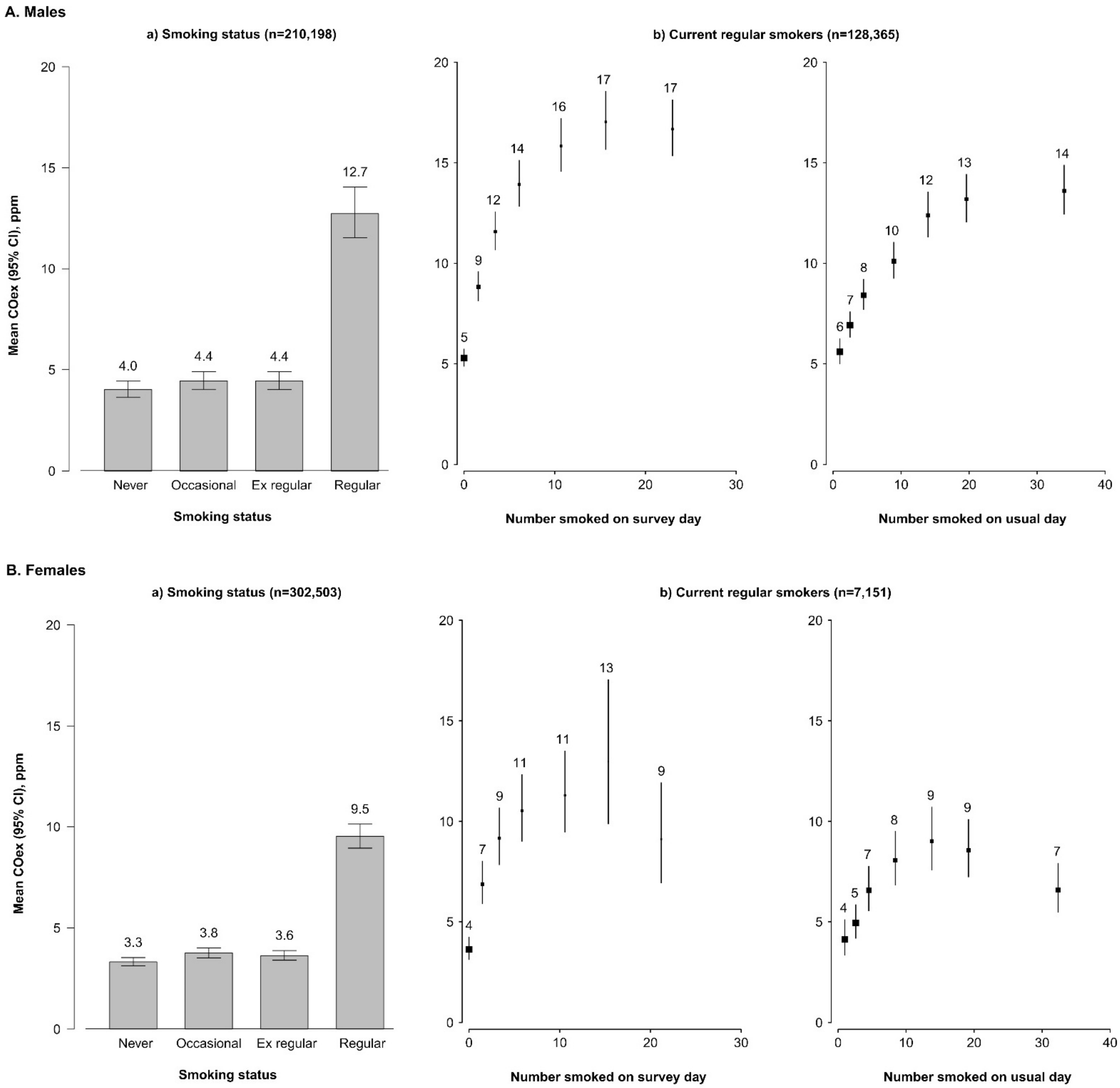

© 2026 Bueno Lopez C et al. *JAMA Neurology*.

eFigure 4: Exhaled CO levels by smoking status and number of cigarettes (or equivalents) smoked per day by urban and rural residence

Conventions as eFigure 3. Means are additionally adjusted for sex.

A. Urban

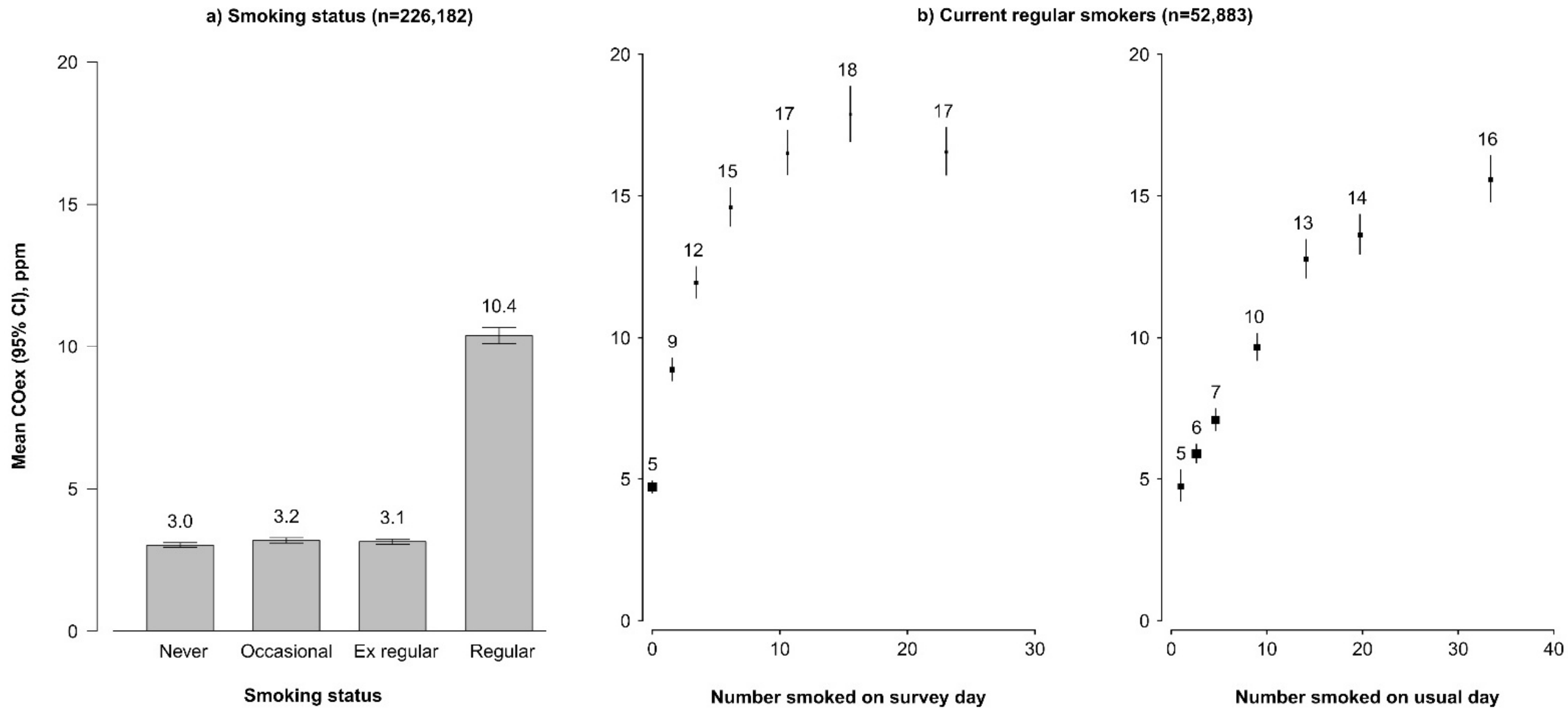

B. Rural

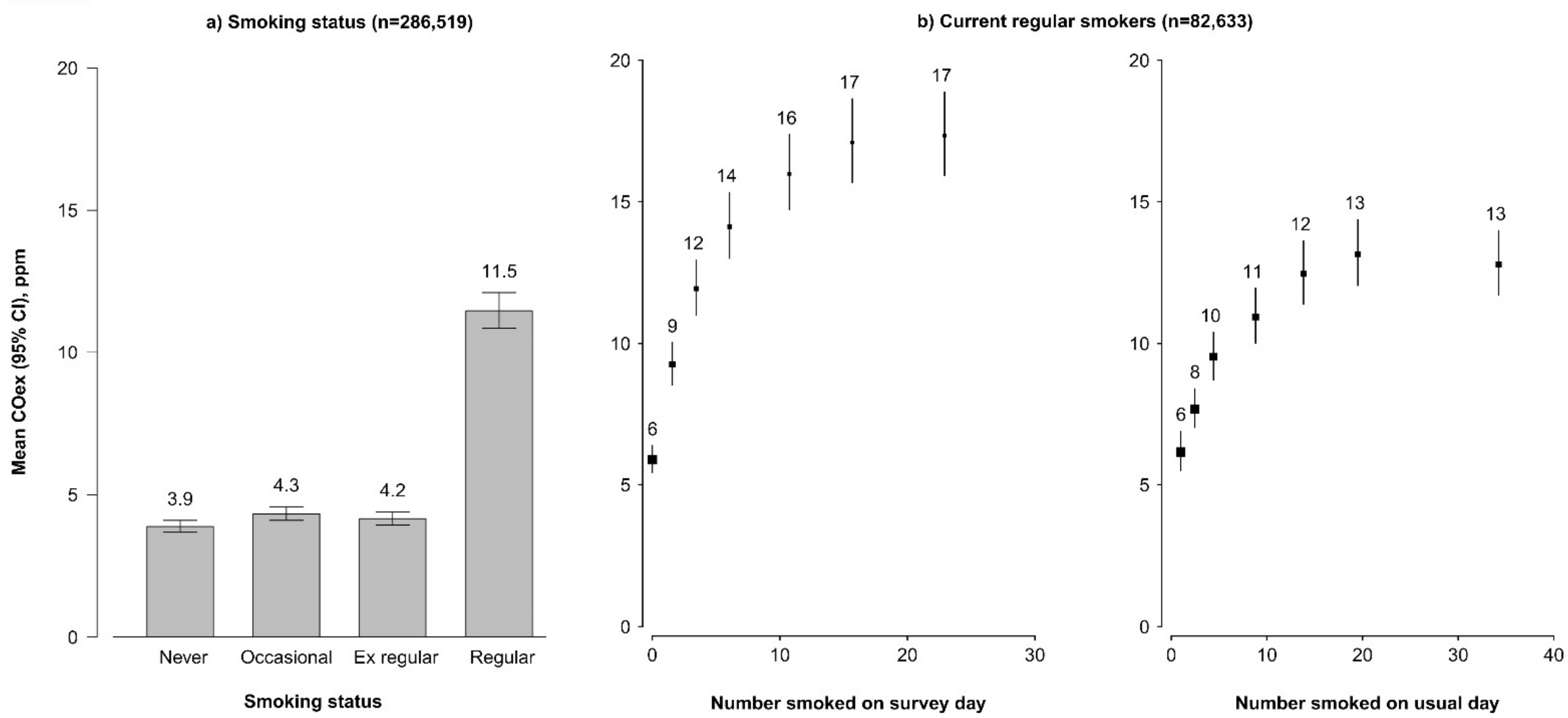

© 2026 Bueno Lopez C et al. *JAMA Neurology*.

eFigure 5: Incidence rates of Parkinson’s disease by age and study area

Error bars represent 95% CIs.

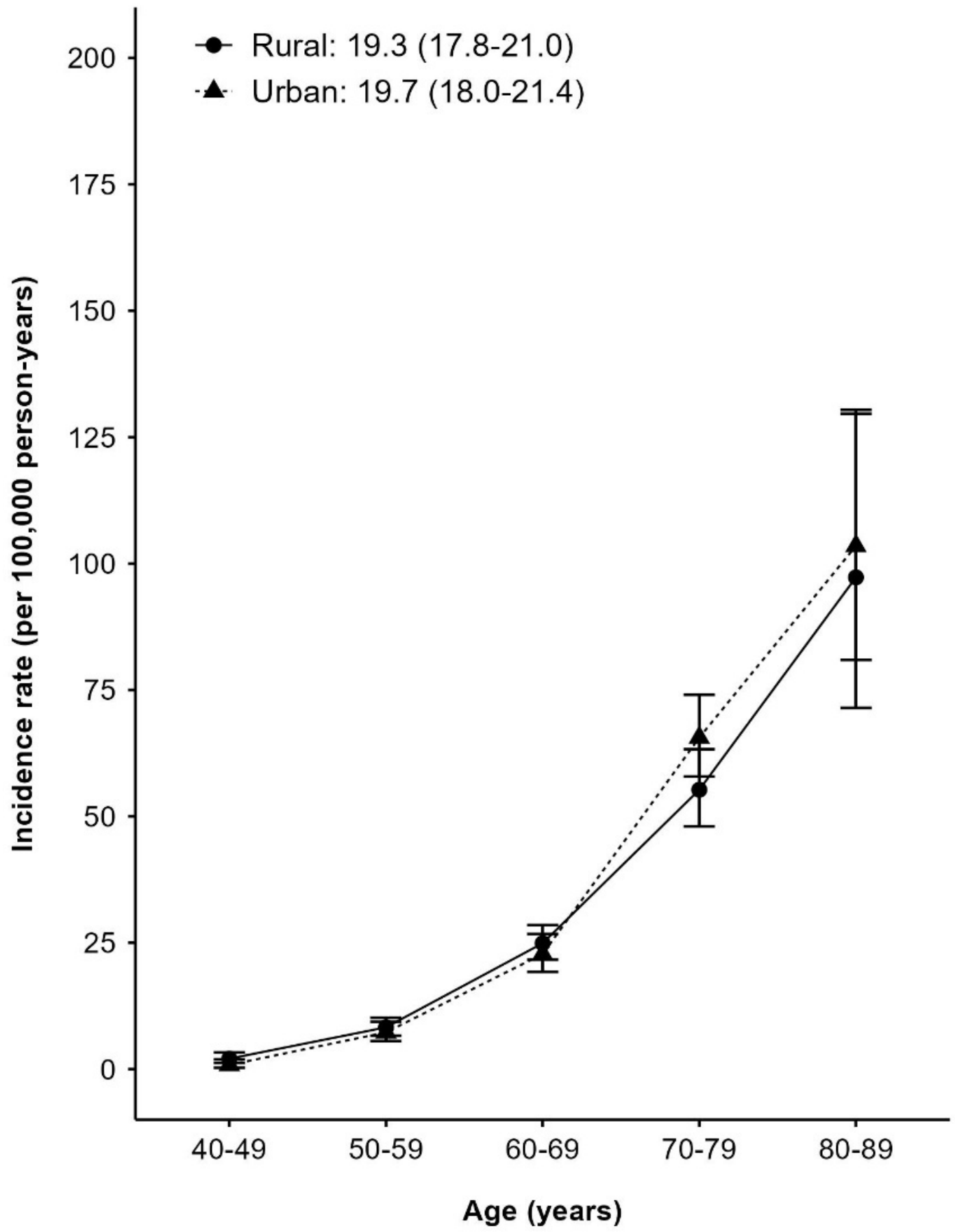

© 2026 Bueno Lopez C et al. *JAMA Neurology*.

eFigure 6: Number of participants with diagnoses of different neurodegenerative diseases

Number of participants with single or multiple neurodegenerative diseases in full cohort. Abbreviations: Parkinsonism: All-cause parkinsonism without a primary PD diagnosis. Vascular=vascular dementia; Alzheimer's=Alzheimer's dementia; AMD=age-related macular degeneration; Other=other/unspecified dementia; PD=Parkinson's disease; Atrophy=brain atrophy.

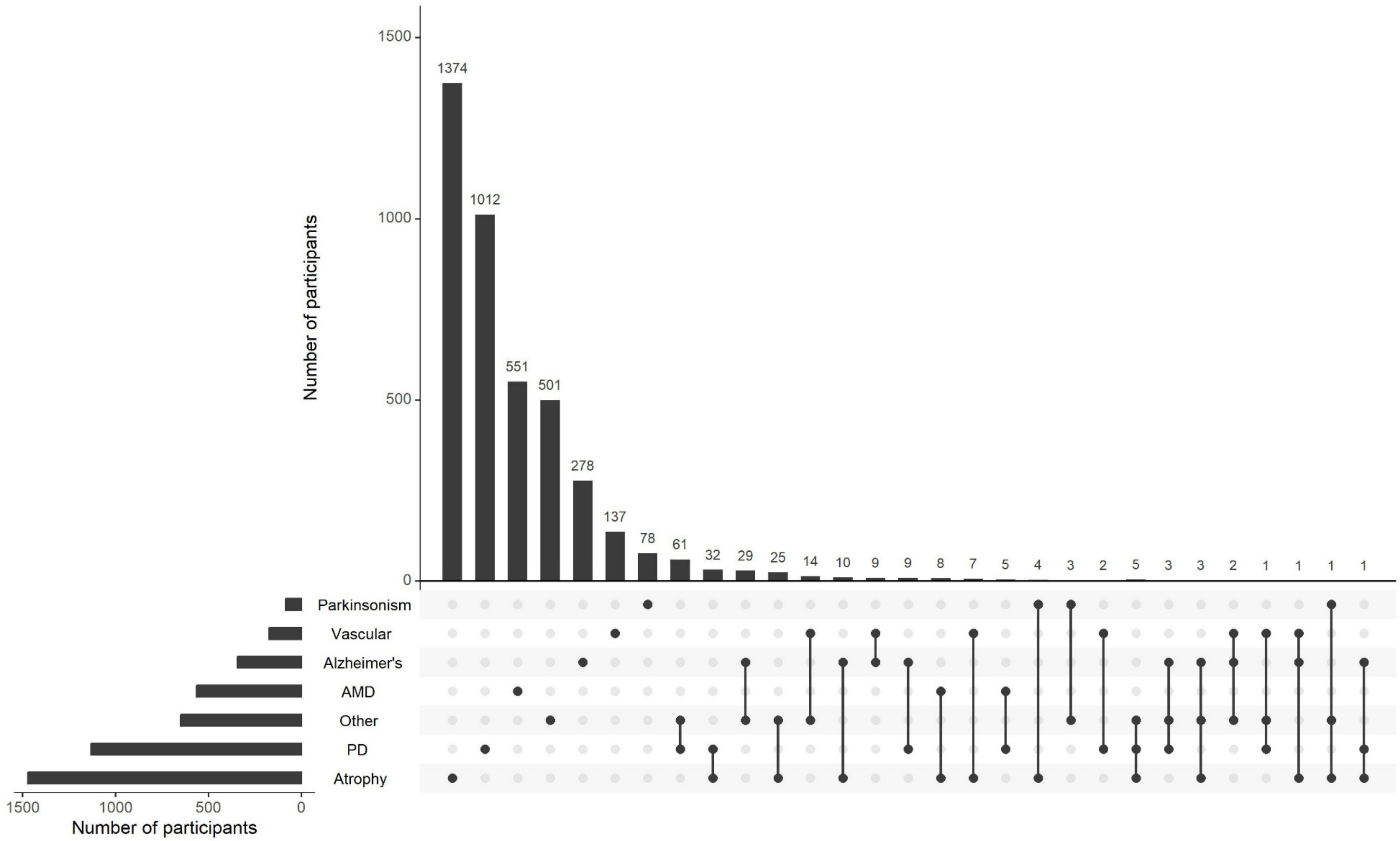

© 2026 Bueno Lopez C et al. *JAMA Neurology*.

eFigure 7: Association of smoking with risk of Parkinson's disease, by different smoking characteristics

The hazard ratios (HRs) are shown as squares and 95% CIs are shown as horizontal lines. HRs are stratified by age-at-risk (5-year groups), sex, and study area and adjusted for education, BMI, alcohol consumption and physical activity. The chi-square and p-values assess evidence of a linear gradient across ordered strata. Mean COex=3.5 ppm and mean COHb=0.6% in never smokers not exposed to passive smoking. COHb=carboxy haemoglobin.

	Mean COex	Mean COHb	No. of events	HR (95% CI)
**Smoking status**				
Never regular	4.7	0.8	766	1.00 (0.88, 1.14)
Former regular	5.1	0.8	63	0.92 (0.72, 1.18)
Regular	13.7	2.2	302	0.70 (0.62, 0.79)
				Heterogeneity: χ^2_2=16.6 (p=2.5e-04)
**Age started smoking, years**				
<20	13.8	2.2	163	0.67 (0.54, 0.83)
20-24	13.2	2.1	59	0.77 (0.57, 1.04)
≥25	11.2	1.8	143	0.77 (0.62, 0.95)
				Trend: χ^2_1=0.8 (p=0.37)
**Level of inhalation**				
Mouth only	10.3	1.7	91	0.65 (0.50, 0.84)
Throat	11.7	1.9	111	0.77 (0.61, 0.98)
Lung	15.0	2.4	163	0.73 (0.59, 0.90)
				Heterogeneity: χ^2_2=0.9 (p=0.62)
**Smoking pack-years**				
<10	10.4	1.7	66	0.82 (0.62, 1.08)
10-24	13.5	2.2	114	0.78 (0.62, 0.98)
≥25	13.8	2.2	180	0.64 (0.52, 0.79)
				Trend: χ^2_1=2.2 (p=0.14)
**Never smokers**				
Passive smoking	4.7	0.8	572	0.90 (0.72, 1.12)

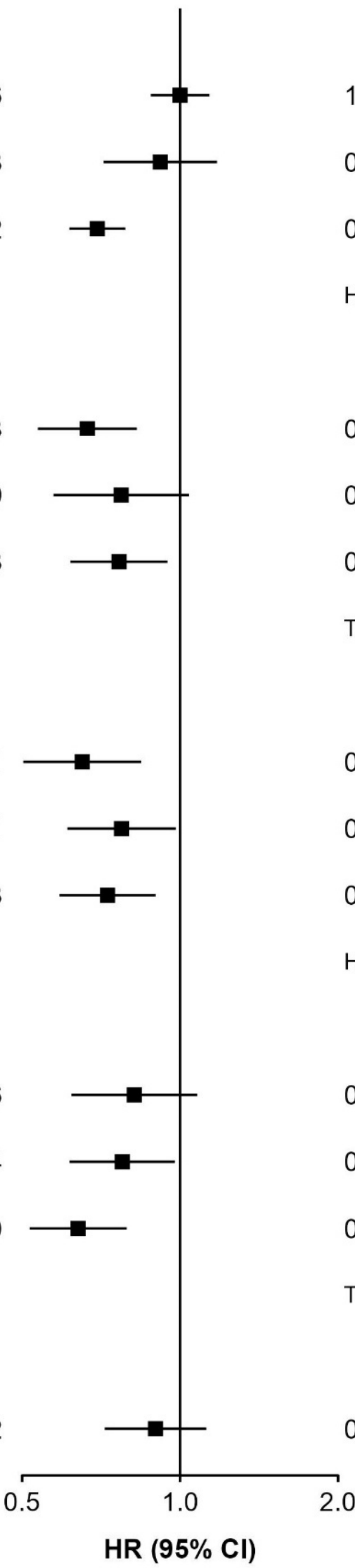

© 2026 Bueno Lopez C et al. *JAMA Neurology*.

eFigure 8: Association of exhaled CO with risk of Parkinson's disease among never smokers and regular smokers, by group specific quintiles

The hazard ratios (HRs) by COex quintiles are shown as squares and 95% CIs are shown as horizontal lines. HRs are stratified by age-at-risk (5-year groups), sex, and study area and adjusted for education, BMI, alcohol consumption, physical activity, season, time of day at survey, solid fuel use, and exposure to passive smoking (among never smokers). The chi-square and p-values assess evidence of a linear gradient across ordered strata. COHb=carboxy haemoglobin.

	Mean cigs/d	Mean COex	Mean COHb	No. of events	HR (95% CI)
**Never smokers**					
**COex, ppm**					
<2.0		1.2	0.2	192	1.00 (0.85, 1.18)
2.0 to <3.0		2.2	0.4	169	0.85 (0.73, 0.99)
3.0 to <4.0		3.2	0.5	96	0.65 (0.54, 0.80)
4.0 to <6.0		4.6	0.7	98	0.62 (0.51, 0.76)
≥6.0		12.4	2.0	120	0.66 (0.53, 0.82)
					Trend: χ_1^2=16.5 (p=4.8e-05)
**Regular smokers**					
**COex, ppm**					
<5.0	17.5	2.8	0.5	99	1.00 (0.80, 1.25)
5.0 to <9.5	16.4	6.9	1.1	85	0.95 (0.77, 1.18)
9.5 to <14.5	16.6	11.7	1.9	45	0.63 (0.47, 0.84)
14.5 to <21.5	18.0	17.4	2.8	34	0.50 (0.36, 0.71)
≥21.5	20.4	30.2	4.8	39	0.62 (0.44, 0.87)
					Trend: χ_1^2=14.3 (p=1.6e-04)

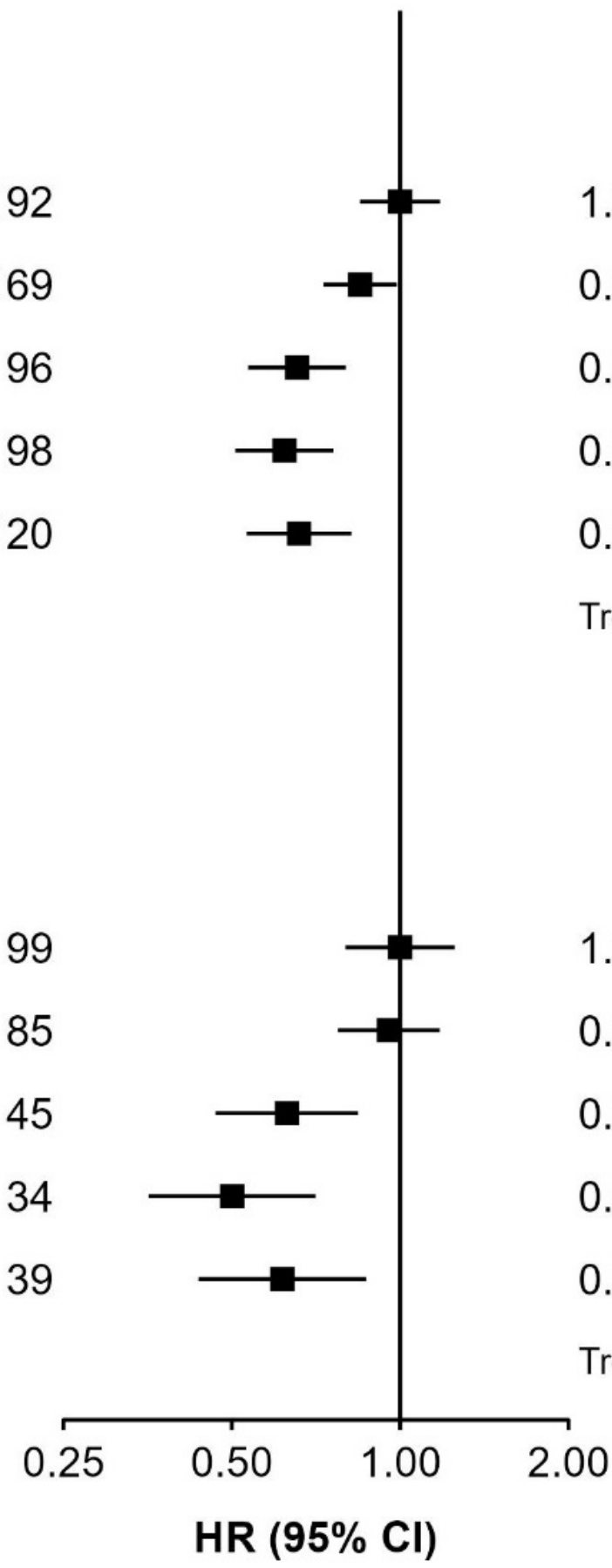

© 2026 Bueno Lopez C et al. *JAMA Neurology*.

eFigure 9: Associations of exhaled CO with risks of selected disease outcomes, applying sequential exclusion criteria

The hazard ratios (HRs) are shown as squares and 95% CIs are shown as horizontal lines. HRs are stratified by age-at-risk (5-year groups), sex, smoking (never/ever regular) and study area and adjusted for education, BMI, alcohol consumption, physical activity, season, time of day at survey, solid fuel use, and exposure to passive smoking. The chi-square and p-values assess evidence of a linear gradient across ordered strata. Baseline disease=self-reported IHD, stroke, cancer, asthma, chronic obstructive pulmonary disease, emphysema, bronchitis, or tuberculosis at recruitment. Abbreviations: IHD=ischaemic heart disease.

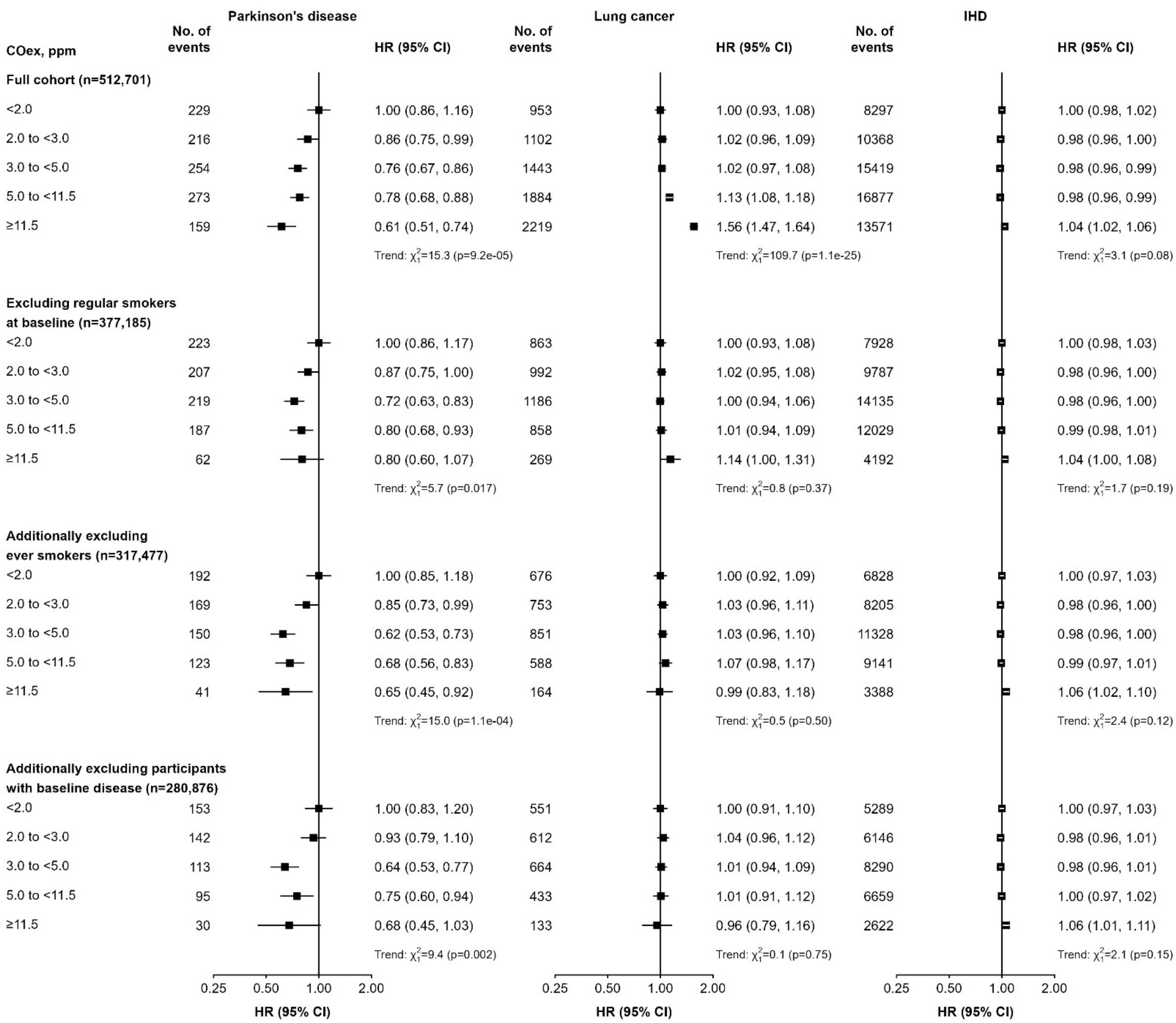

COex, ppm	Parkinson's disease: No. of events	Parkinson's disease: HR (95% CI)	Lung cancer: No. of events	Lung cancer: HR (95% CI)	IHD: No. of events	IHD: HR (95% CI)
**Full cohort (n=512,701)**						
<2.0	229	1.00 (0.86, 1.16)	953	1.00 (0.93, 1.08)	8297	1.00 (0.98, 1.02)
2.0 to <3.0	216	0.86 (0.75, 0.99)	1102	1.02 (0.96, 1.09)	10368	0.98 (0.96, 1.00)
3.0 to <5.0	254	0.76 (0.67, 0.86)	1443	1.02 (0.97, 1.08)	15419	0.98 (0.96, 0.99)
5.0 to <11.5	273	0.78 (0.68, 0.88)	1884	1.13 (1.08, 1.18)	16877	0.98 (0.96, 0.99)
≥11.5	159	0.61 (0.51, 0.74)	2219	1.56 (1.47, 1.64)	13571	1.04 (1.02, 1.06)
		Trend: χ^2_1=15.3 (p=9.2e-05)		Trend: χ^2_1=109.7 (p=1.1e-25)		Trend: χ^2_1=3.1 (p=0.08)
**Excluding regular smokers at baseline (n=377,185)**						
<2.0	223	1.00 (0.86, 1.17)	863	1.00 (0.93, 1.08)	7928	1.00 (0.98, 1.03)
2.0 to <3.0	207	0.87 (0.75, 1.00)	992	1.02 (0.95, 1.08)	9787	0.98 (0.96, 1.00)
3.0 to <5.0	219	0.72 (0.63, 0.83)	1186	1.00 (0.94, 1.06)	14135	0.98 (0.96, 1.00)
5.0 to <11.5	187	0.80 (0.68, 0.93)	858	1.01 (0.94, 1.09)	12029	0.99 (0.98, 1.01)
≥11.5	62	0.80 (0.60, 1.07)	269	1.14 (1.00, 1.31)	4192	1.04 (1.00, 1.08)
		Trend: χ^2_1=5.7 (p=0.017)		Trend: χ^2_1=0.8 (p=0.37)		Trend: χ^2_1=1.7 (p=0.19)
**Additionally excluding ever smokers (n=317,477)**						
<2.0	192	1.00 (0.85, 1.18)	676	1.00 (0.92, 1.09)	6828	1.00 (0.97, 1.03)
2.0 to <3.0	169	0.85 (0.73, 0.99)	753	1.03 (0.96, 1.11)	8205	0.98 (0.96, 1.00)
3.0 to <5.0	150	0.62 (0.53, 0.73)	851	1.03 (0.96, 1.10)	11328	0.98 (0.96, 1.00)
5.0 to <11.5	123	0.68 (0.56, 0.83)	588	1.07 (0.98, 1.17)	9141	0.99 (0.97, 1.01)
≥11.5	41	0.65 (0.45, 0.92)	164	0.99 (0.83, 1.18)	3388	1.06 (1.02, 1.10)
		Trend: χ^2_1=15.0 (p=1.1e-04)		Trend: χ^2_1=0.5 (p=0.50)		Trend: χ^2_1=2.4 (p=0.12)
**Additionally excluding participants with baseline disease (n=280,876)**						
<2.0	153	1.00 (0.83, 1.20)	551	1.00 (0.91, 1.10)	5289	1.00 (0.97, 1.03)
2.0 to <3.0	142	0.93 (0.79, 1.10)	612	1.04 (0.96, 1.12)	6146	0.98 (0.96, 1.01)
3.0 to <5.0	113	0.64 (0.53, 0.77)	664	1.01 (0.94, 1.09)	8290	0.98 (0.96, 1.01)
5.0 to <11.5	95	0.75 (0.60, 0.94)	433	1.01 (0.91, 1.12)	6659	1.00 (0.97, 1.02)
≥11.5	30	0.68 (0.45, 1.03)	133	0.96 (0.79, 1.16)	2622	1.06 (1.01, 1.11)
		Trend: χ^2_1=9.4 (p=0.002)		Trend: χ^2_1=0.1 (p=0.75)		Trend: χ^2_1=2.1 (p=0.15)

© 2026 Bueno Lopez C et al. *JAMA Neurology*.

eFigure 10: Association of exhaled CO with risk of Parkinson's disease among never smokers, excluding participants from Henan

The hazard ratios (HRs) are shown as squares (scaled proportional to the variance of the log risk) and 95% CIs are shown as horizontal lines. HRs are stratified by age-at-risk (5-year groups), sex, and study area and adjusted for education, BMI, alcohol consumption, physical activity, season, time of day at survey, solid fuel use, and exposure to passive smoking. The chi-square and p-values assess evidence of a linear gradient across ordered strata. COHb=carboxy haemoglobin.

COex, ppm	Mean COex	Mean COHb	No. of events	HR (95% CI)
<2.0	1.2	0.2	192	1.00 (0.85, 1.18)
2.0 to <3.0	2.2	0.4	165	0.84 (0.72, 0.98)
3.0 to <5.0	3.5	0.6	141	0.61 (0.52, 0.72)
5.0 to <11.5	6.6	1.1	99	0.67 (0.55, 0.83)
≥11.5	17.1	2.7	11	0.44 (0.24, 0.80)

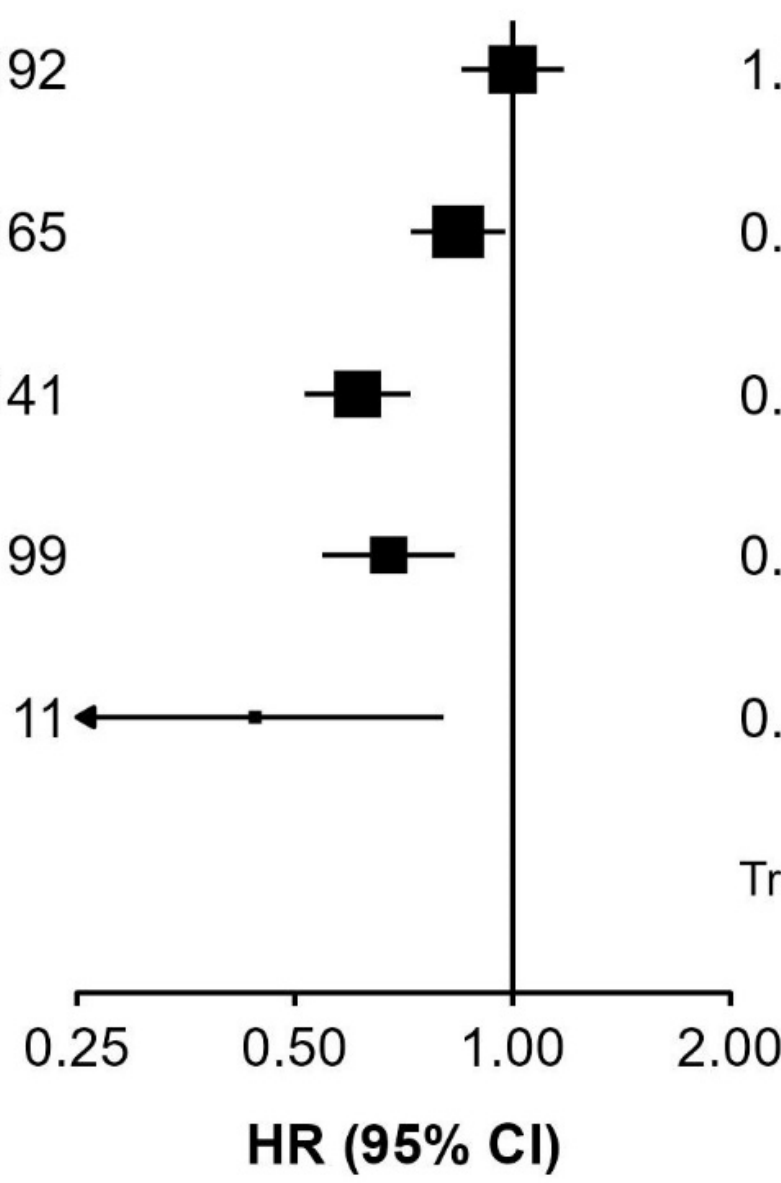

Trend: χ^2_1=19.0 (p=1.3e-05)

© 2026 Bueno Lopez C et al. *JAMA Neurology*.

eFigure 11: Association of smoking and exhaled CO with risk of Parkinsonism

The hazard ratios (HRs) are shown as squares and 95% CIs are shown as horizontal lines. HRs are stratified by age-at-risk (5-year groups), sex, and study area and adjusted for education, BMI, alcohol consumption and physical activity. Associations with COex were additionally adjusted for season, time of day at survey, solid fuel use, and exposure to passive smoking. The chi-square and p-values assess evidence of a linear gradient across ordered strata. COHb=carboxy haemoglobin.

	Mean COex	Mean COHb	No. of events	HR (95% CI)
**Smoking categories**				
Never regular	4.7	0.8	819	1.00 (0.88, 1.14)
Former regular	5.1	0.8	66	0.89 (0.70, 1.14)
Regular, <15 cigs/d	11.8	1.9	149	0.81 (0.69, 0.95)
15-24 cigs/d	14.9	2.4	122	0.60 (0.50, 0.72)
≥25 cigs/d	14.9	2.4	60	0.71 (0.54, 0.92)
				Trend: χ^2_1=18.4 (p=1.8e-05)
**Never smokers**				
**COex, ppm**				
<2.0	1.2	0.2	203	1.00 (0.85, 1.17)
2.0 to <3.0	2.2	0.4	181	0.85 (0.74, 0.99)
3.0 to <5.0	3.6	0.6	159	0.62 (0.53, 0.72)
5.0 to <11.5	6.8	1.1	136	0.70 (0.58, 0.83)
≥11.5	20.7	3.3	42	0.61 (0.43, 0.87)
				Trend: χ^2_1=16.6 (p=4.6e-05)

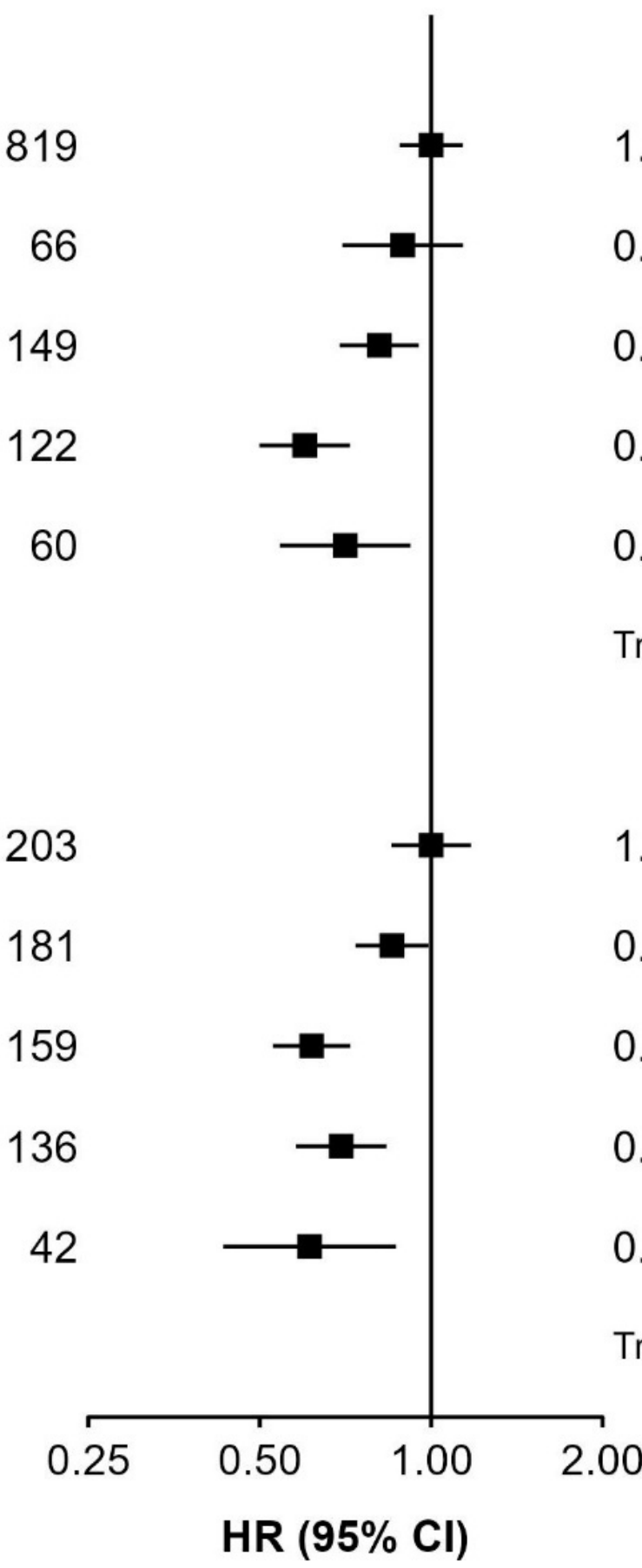

© 2026 Bueno Lopez C et al. *JAMA Neurology*.

eFigure 12: Adjusted HRs for risk of Parkinson's disease associated with high exhaled CO (≥3 ppm vs. <3 ppm) among never smokers, in various participant subgroups

The hazard ratios (HRs) are shown as squares (scaled proportional to the variance of the log risk) and 95% CIs are shown as horizontal lines. HRs are stratified by age-at-risk (5-year groups), sex, and study area and adjusted for education, BMI, alcohol consumption, physical activity, season, time of day at survey, solid fuel use, and exposure to passive smoking. The chi-square and p-values are shown for tests of trend and heterogeneity between subgroups. Solid fuel=coal or wood. Abbreviations: MET=metabolic equivalent of task.

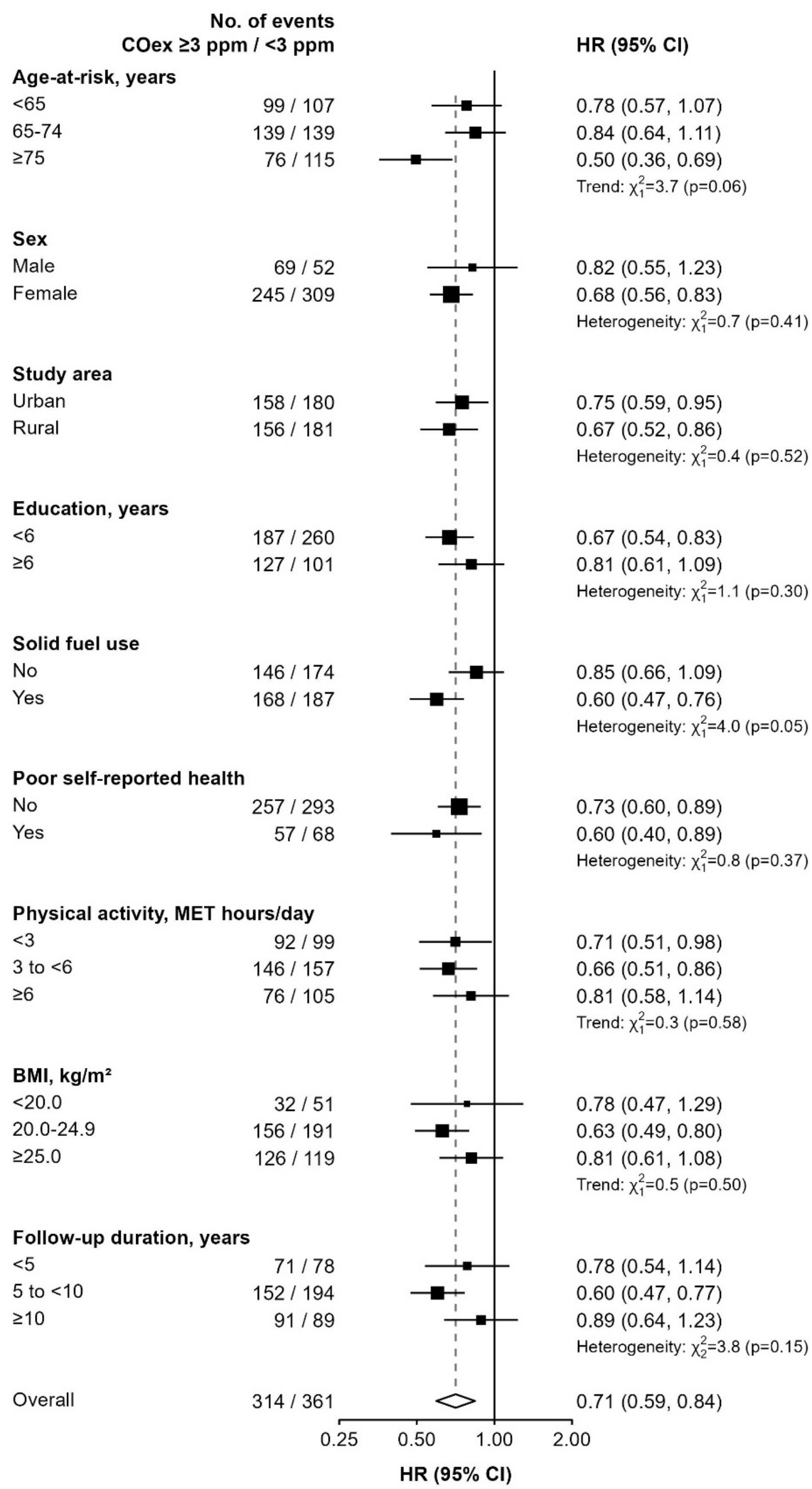

© 2026 Bueno Lopez C et al. *JAMA Neurology*.

eFigure 13: Association of exhaled CO with risk of Parkinson's disease among never smokers, by study area

The hazard ratios (HRs) are shown as squares (scaled proportional to the variance of the log risk) and 95% CIs are shown as horizontal lines. HRs are stratified by age-at-risk (5-year groups) and sex and adjusted for education. The chi-square and p-values are shown for tests of heterogeneity between subgroups.

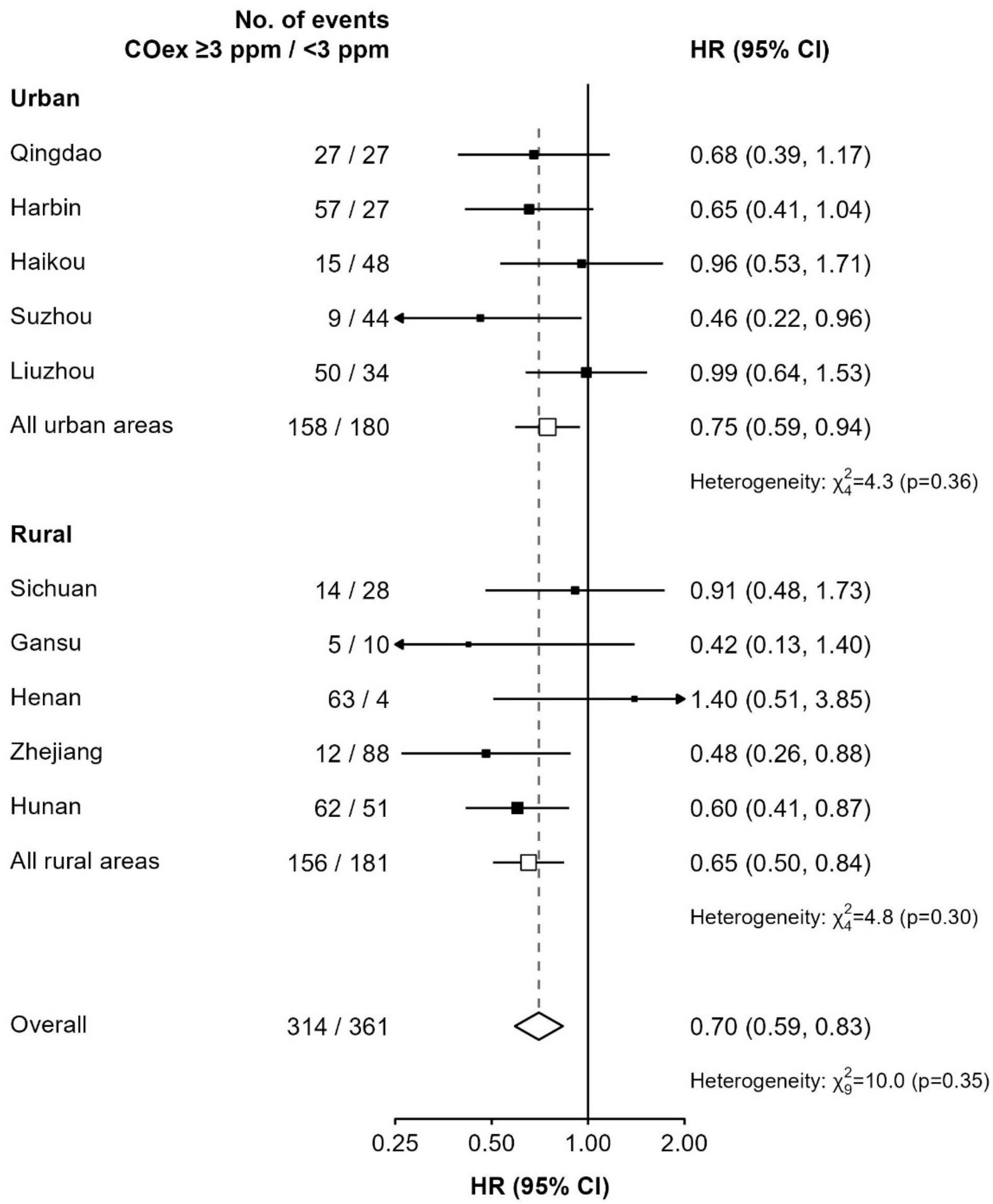

© 2026 Bueno Lopez C et al. *JAMA Neurology*.

eFigure 14: Association of exhaled CO with risk of Parkinson’s disease among never smokers, by sex and urban/rural residence

Conventions as eFigure 10.

COex, ppm	Mean COex	Mean COHb	No. of events	HR (95% CI)
**Males**				
<2.0	1.3	0.2	24	1.00 (0.64, 1.57)
2.0 to <3.0	2.2	0.4	28	0.91 (0.62, 1.33)
3.0 to <5.0	3.6	0.6	31	0.67 (0.47, 0.95)
5.0 to <11.5	6.7	1.1	30	0.92 (0.62, 1.35)
≥11.5	18.9	3.0	8	1.08 (0.50, 2.34)
				Trend: χ^2_1=0.0 (p=0.84)
**Females**				
<2.0	1.2	0.2	168	1.00 (0.84, 1.19)
2.0 to <3.0	2.2	0.4	141	0.84 (0.71, 0.99)
3.0 to <5.0	3.6	0.6	119	0.62 (0.51, 0.74)
5.0 to <11.5	6.9	1.1	93	0.63 (0.51, 0.79)
≥11.5	20.9	3.4	33	0.57 (0.38, 0.85)
				Trend: χ^2_1=17.6 (p=2.7e-05)
**Rural**				
<2.0	1.2	0.2	111	1.00 (0.80, 1.25)
2.0 to <3.0	2.2	0.4	70	0.74 (0.58, 0.94)
3.0 to <5.0	3.6	0.6	62	0.57 (0.45, 0.73)
5.0 to <11.5	7.0	1.1	61	0.57 (0.44, 0.76)
≥11.5	21.7	3.5	33	0.59 (0.39, 0.90)
				Trend: χ^2_1=11.8 (p=6.0e-04)
**Urban**				
<2.0	1.3	0.2	81	1.00 (0.78, 1.28)
2.0 to <3.0	2.2	0.4	99	0.99 (0.82, 1.21)
3.0 to <5.0	3.5	0.6	88	0.70 (0.57, 0.87)
5.0 to <11.5	6.6	1.1	62	0.83 (0.64, 1.09)
≥11.5	16.3	2.6	8	0.76 (0.38, 1.54)
				Trend: χ^2_1=3.4 (p=0.066)

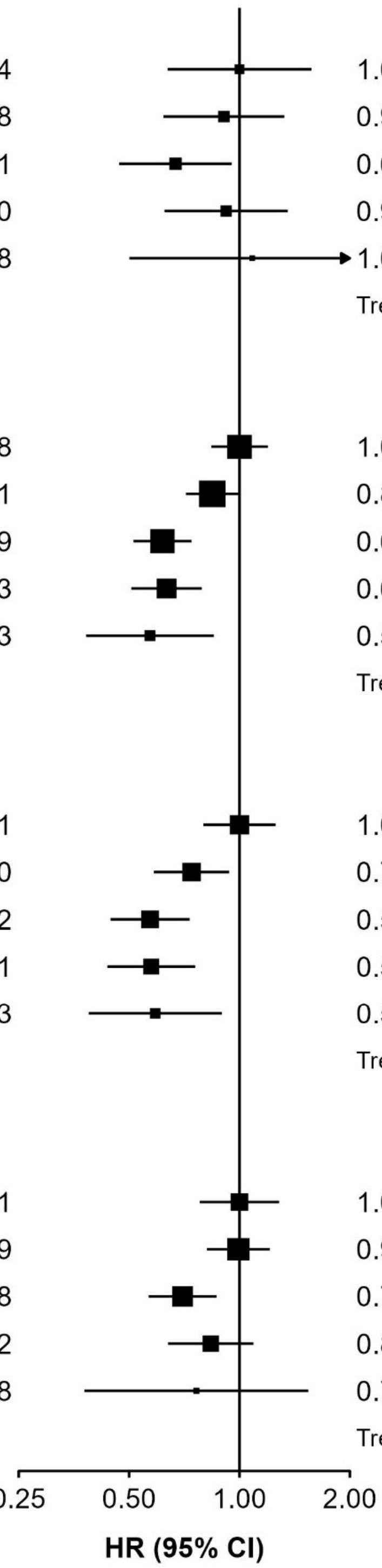

© 2026 Bueno Lopez C et al. *JAMA Neurology*.

eFigure 15: Association of exhaled CO with risk of Parkinson's disease by smoking characteristics, in males

*Additionally stratified by smoking (never/ever regular). Regular smokers=participants who reported smoking daily or almost every day or who stopped for illness. Conventions as eFigure 12.

	No. of events COex ≥3 ppm / <3 ppm	HR (95% CI)
Full cohort* (n=210,198)	419 / 124	1.02 (0.81, 1.27)
Not regular smokers at baseline* (n=67,995)	175 / 88	1.10 (0.82, 1.46)
Never regular smokers (n=53,916)	125 / 76	0.90 (0.65, 1.23)
Smoked <100cigs/lifetime (n=36,806)	80 / 56	0.80 (0.54, 1.17)
Never smokers (n=30,302)	69 / 52	0.82 (0.55, 1.23)

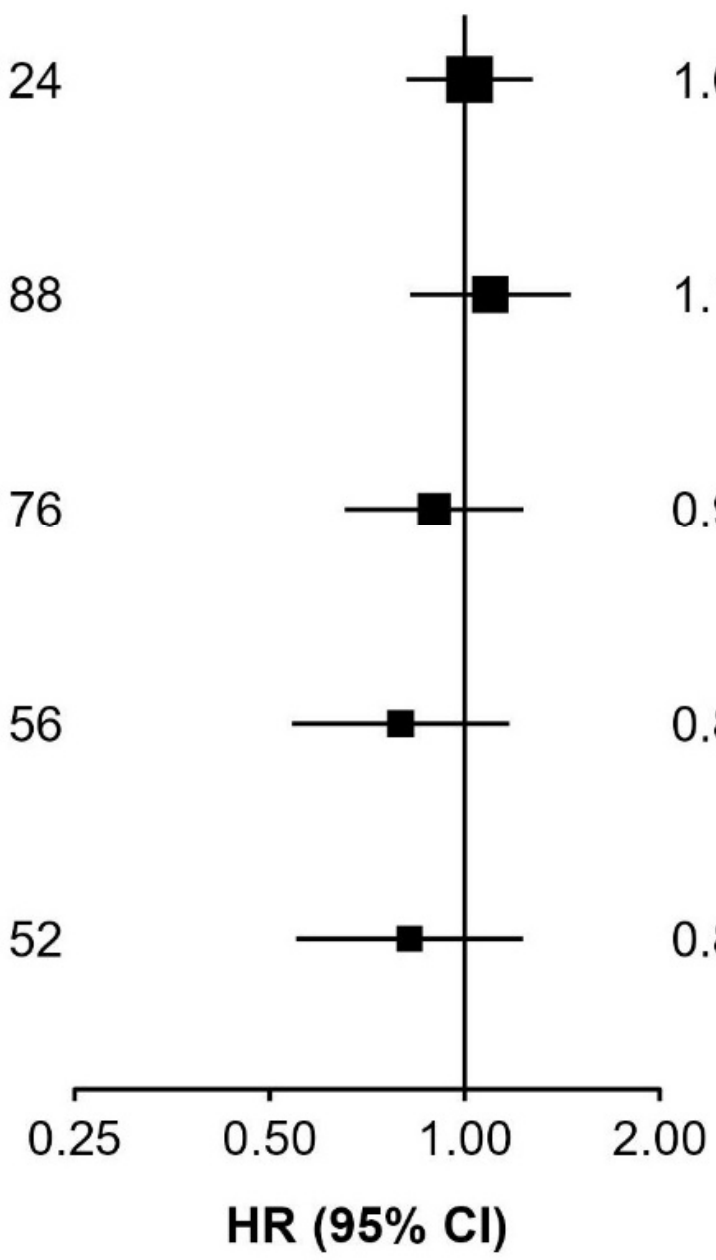

© 2026 Bueno Lopez C et al. *JAMA Neurology*.

eFigure 16: Association of smoking and exhaled CO with risk of brain atrophy and macular degeneration

The hazard ratios (HRs) are shown as squares and 95% CIs are shown as horizontal lines. HRs are stratified by age-at-risk (5-year groups), sex, and study area and adjusted for education, BMI, alcohol consumption and physical activity. Associations with COex were additionally adjusted for season, time of day at survey, solid fuel use, and exposure to passive smoking. The chi-square and p-values assess evidence of a linear gradient across ordered strata. COHb=carboxy haemoglobin.

Ever-regular smoking was associated with higher risk of macular degeneration in males (HR 1.45 [95% CI 1.03-2.03]).

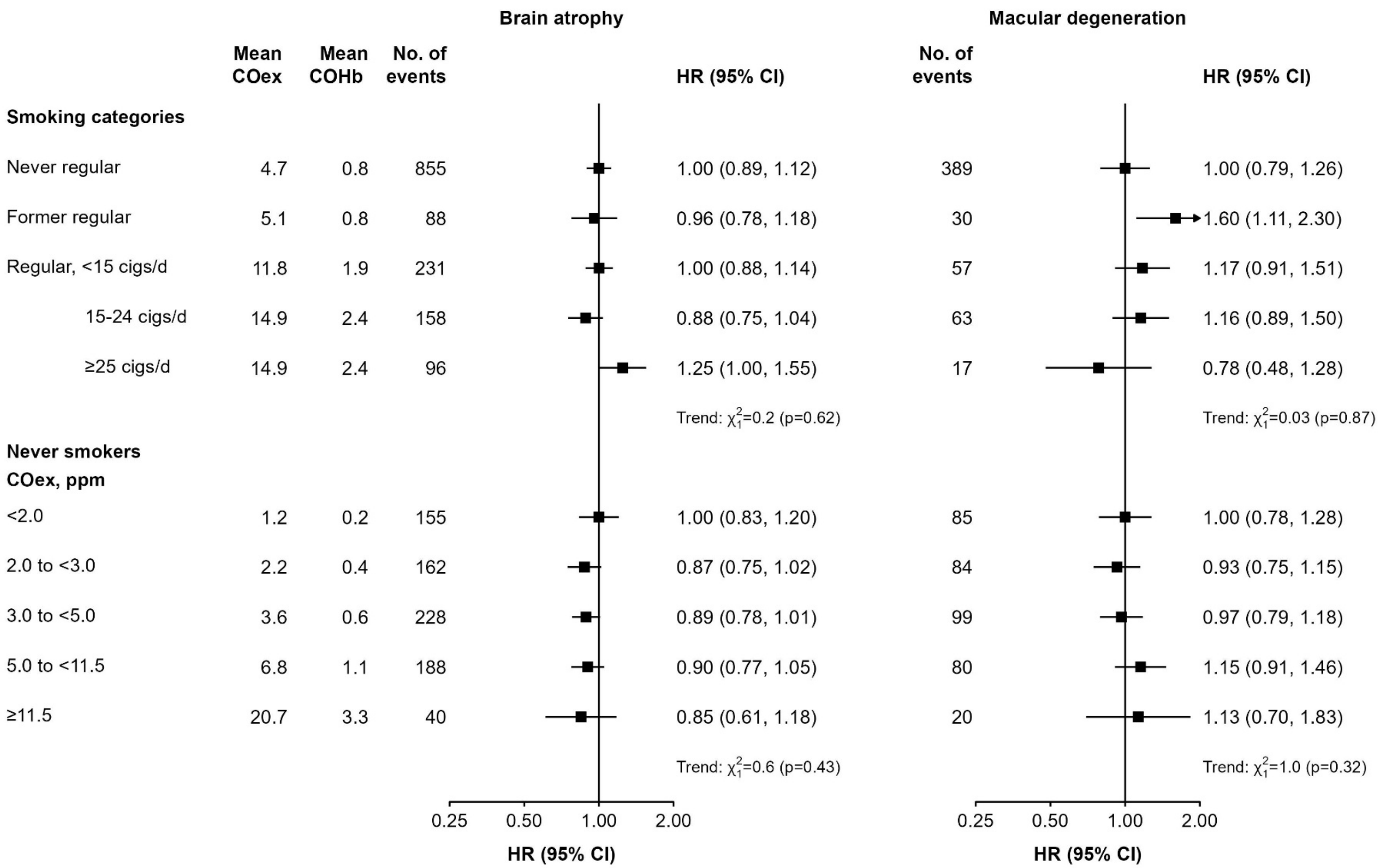

© 2026 Bueno Lopez C et al. *JAMA Neurology*.

eFigure 17: Association of smoking and exhaled CO with risk of dementia, by disease subtypes

Conventions as eFigure 16.

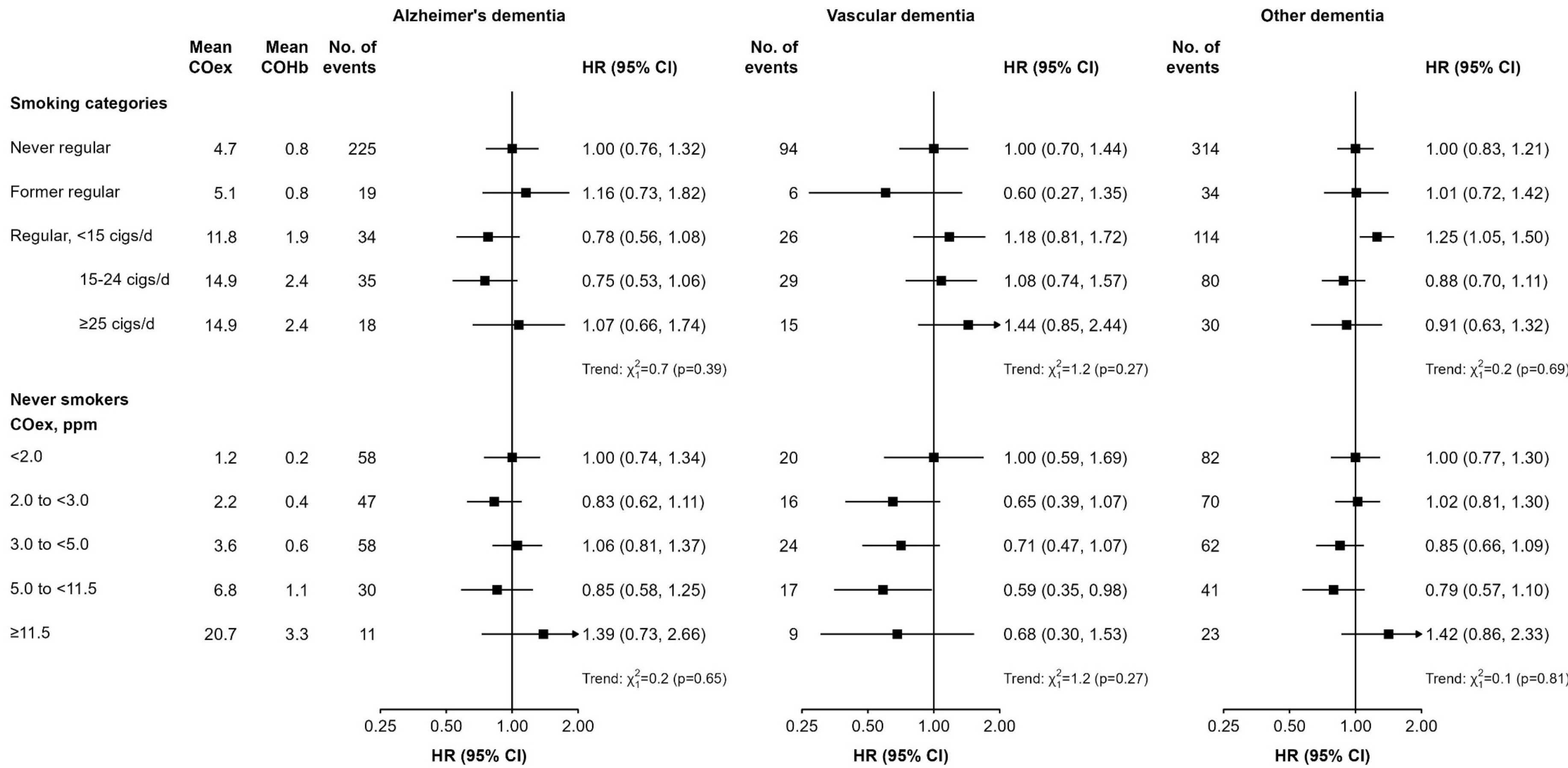

© 2026 Bueno Lopez C et al. *JAMA Neurology*.

eFigure 18: Association of smoking and exhaled CO with risks of other neurodegenerative diseases, without excluding co-diagnoses of Parkinsonism

Conventions as eFigure 16.

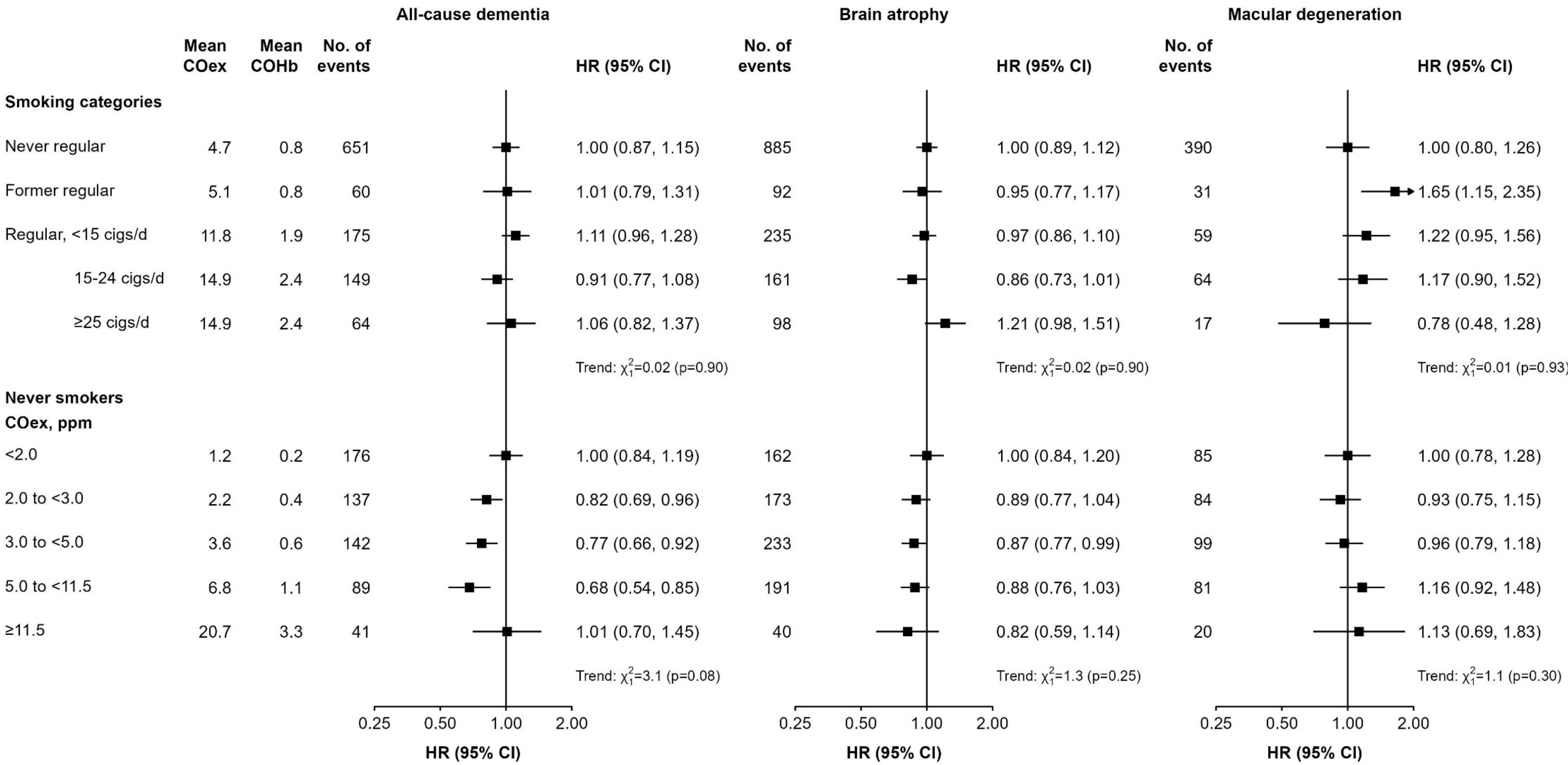

© 2026 Bueno Lopez C et al. *JAMA Neurology*.

eFigure 19: Association of smoking and exhaled CO with risks of dementia subtypes, without excluding co-diagnoses of Parkinsonism

Conventions as eFigure 16.

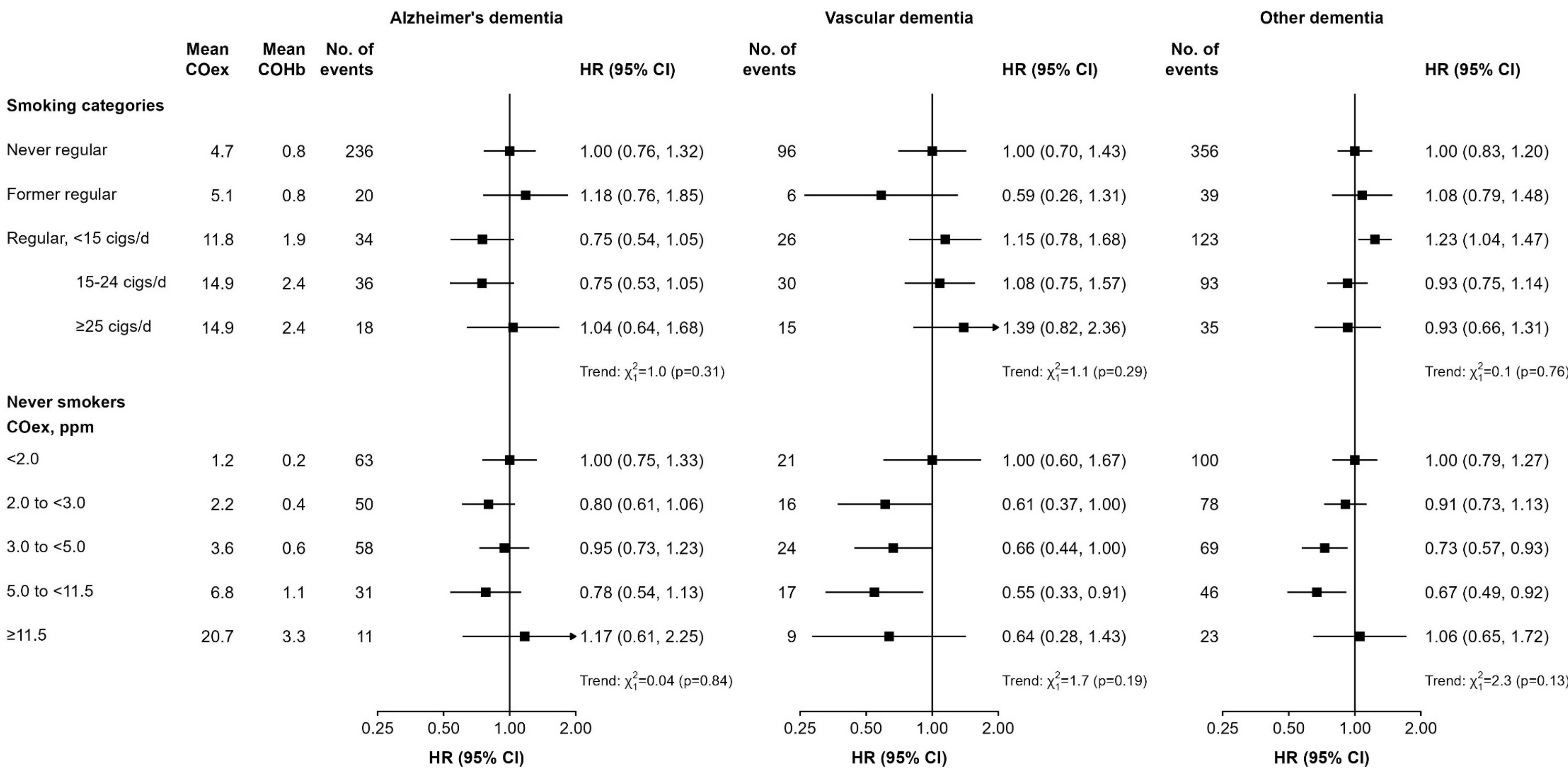

© 2026 Bueno Lopez C et al. *JAMA Neurology*.

eFigure 20: Association of smoking and exhaled CO with risks of stroke and all-cause mortality

Conventions as eFigure 16.

	Mean COex	Mean COHb	Stroke: No. of events	Stroke: HR (95% CI)	All-cause mortality: No. of events	All-cause mortality: HR (95% CI)
**Smoking categories**						
Never regular	4.7	0.8	44269	1.00 (0.98, 1.02)	29221	1.00 (0.98, 1.02)
Former regular	5.1	0.8	2909	0.98 (0.94, 1.02)	2609	1.08 (1.04, 1.12)
Regular, <15 cigs/d	11.8	1.9	9583	1.12 (1.10, 1.14)	10342	1.30 (1.28, 1.33)
15-24 cigs/d	14.9	2.4	9207	1.17 (1.14, 1.19)	9900	1.36 (1.33, 1.39)
≥25 cigs/d	14.9	2.4	3697	1.26 (1.22, 1.31)	4251	1.49 (1.45, 1.54)
				Trend: χ^2_1=218.0 (p=2.4e-49)		Trend: χ^2_1=654.6 (p=2.3e-144)
**Never smokers COex, ppm**						
<2.0	1.2	0.2	7934	1.00 (0.97, 1.03)	6207	1.00 (0.97, 1.03)
2.0 to <3.0	2.2	0.4	8392	1.01 (0.99, 1.03)	5743	0.95 (0.93, 0.98)
3.0 to <5.0	3.6	0.6	10802	1.02 (1.00, 1.04)	6526	0.94 (0.91, 0.96)
5.0 to <11.5	6.8	1.1	9404	1.01 (0.98, 1.03)	5329	0.94 (0.91, 0.96)
≥11.5	20.7	3.3	3788	1.01 (0.98, 1.05)	2140	0.95 (0.91, 1.00)
				Trend: χ^2_1=0.2 (p=0.66)		Trend: χ^2_1=8.7 (p=0.003)

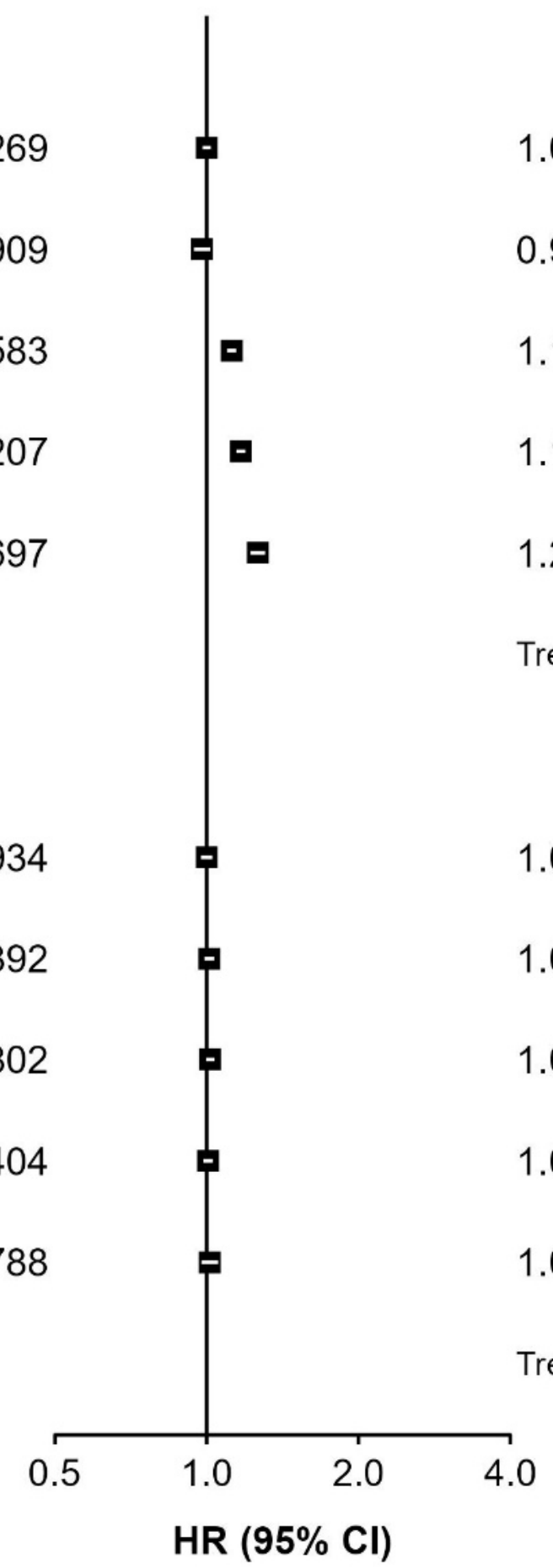

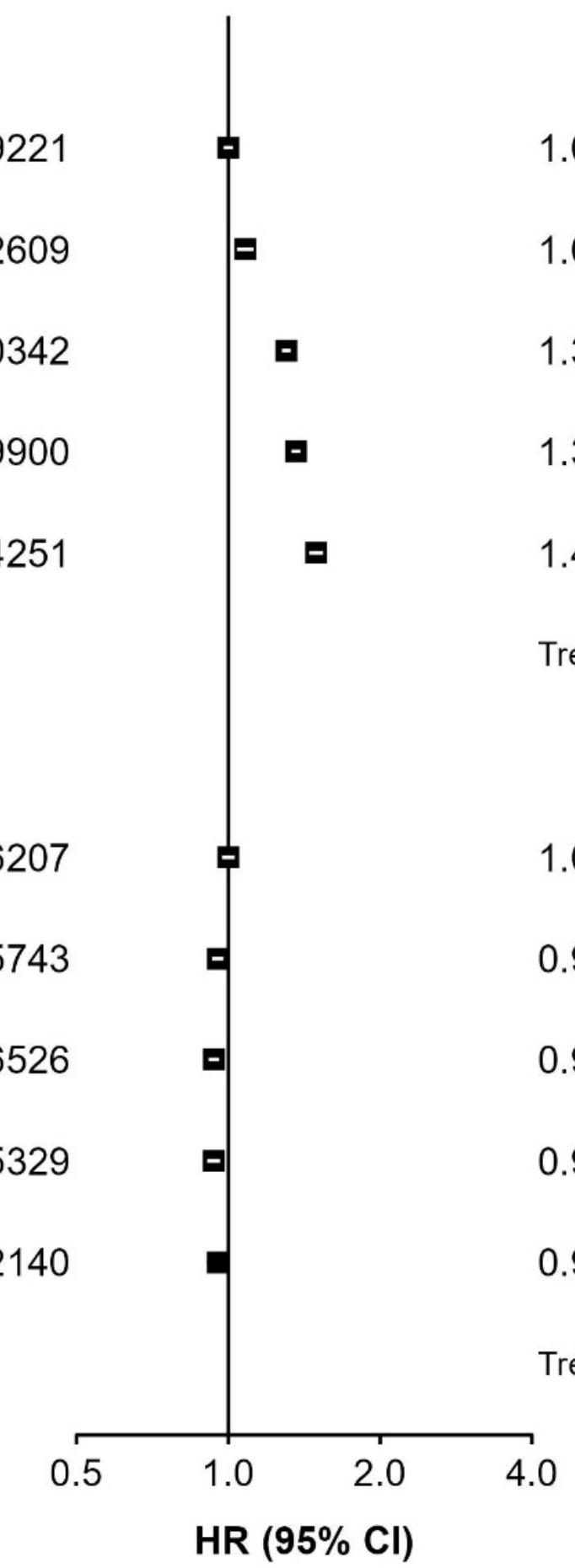

© 2026 Bueno Lopez C et al. *JAMA Neurology*.
