# Supplementary material for: Smoking, Exhaled Carbon Monoxide, and Risk of Parkinson Disease
Source: JAMA Neurol. 2026 Sep 8:e263037. Online ahead of print. doi: 10.1001/jamaneurol.2026.3037 (PMC13555506; doi:10.1001/jamaneurol.2026.3037)
Supplement: Supplementary file 2 [file jamaneurol-e263037-s002.pdf]

Supplemental Online Content: Nonauthor Collaborators
*First name, last name, and suffix (if applicable) are required and will appear in PubMed.

***Group Name(s): China Kadoorie Biobank (CKB) Collaborative Group**							
***First Name and Middle Initial(s)**	***Last Name**	***Suffix (eg, Jr, III)**	Academic Degrees	Institution	Location (city, state/province, country)	Role or Contribution, eg, chair, principal investigator	Group (if more than 1 Group listed in the byline) and/or Subgroup (eg, Steering Committee)
Junshi	Chen						
Zhengming	Chen						
Robert	Clarke						
Rory	Collins						
Liming	Li						
Jun	Lv						
Richard	Peto						
Robin	Walters						
Daniel	Avery						
Maxim	Bernard						
Derrick	Bennett						
Ruth	Boxall						
Ka Hung	Chan						
Yiping	Chen						
Jonathan	Clarke						
Huaidong	Du						
Ahmed Edris	Mohamed						
Hannah	Fry						
Pek Kei	Im						
Andri	Iona						
Christiana	Kartsonaki						
Hubert	Lam						
Kuang	Lin						
Iona	Millwood						
Sam	Morris						
Qunhua	Nie						
Alfred	Pozarickij						
Maryam	Rahmati						
Paul	Ryder						

Supplemental Online Content: Nonauthor Collaborators
*First name, last name, and suffix (if applicable) are required and will appear in PubMed.

***First Name and Middle Initial(s)**	***Last Name**	***Suffix (eg, Jr, III)**	Academic Degrees	Institution	Location (city, state/province, country)	Role or Contribution, eg, chair, principal investigator	Group (if more than 1 Group listed in the byline) and/or Subgroup (eg, Steering Committee)
Dan	Schmidt						
Becky	Stevens						
Lin	Wang						
Neil	Wright						
Ling	Yang						
Xiaoming	Yang						
Pang	Yao						
Yani	Huang						
Sara	Perez Vizan						
Thu	Pham						
Roshi	Shrestha						
Xiao	Han						
Can	Hou						
Qingmei	Xia						
Chao	Liu						
Pei	Pei						
Dianjanyi	Sun						
Canqing	Yu						
Yuanjie	Pang						
Yuting	Han						
Honglu	Bian						
Xinxin	Chen						
Lang	Pan						
Zengchang	Pang						
Ruqin	Gao						
Kunzheng	Lv						
Shanpeng	Li						
Haiping	Duan						
Shaojie	Wang						
Yongmei	Liu						

Supplemental Online Content: Nonauthor Collaborators
*First name, last name, and suffix (if applicable) are required and will appear in PubMed.

***First Name and Middle Initial(s)**	***Last Name**	***Suffix (eg, Jr, III)**	Academic Degrees	Institution	Location (city, state/province, country)	Role or Contribution, eg, chair, principal investigator	Group (if more than 1 Group listed in the byline) and/or Subgroup (eg, Steering Committee)
Ranran	Du						
Liang	Cheng						
Xiaocao	Tian						
Hua	Zhang						
Dan	Hu						
Xiaoyan	Zheng						
Yujie	Wang						
Wei	Sun						
Shichun	Yan						
Yong	Zhou						
Chi	Wang						
Zhenyuan	Wu						
Lishun	Zhai						
Zhaoxi	Pang						
Shiwen	Dong						
Li	Liu						
Dapeng	Yin						
Bin	He						
Ying	Liu						
Xingren	Wang						
Tingting	Ou						
Xiangyang	Zheng						
Dewei	Zheng						
Shuai	Yang						
Lihui	Li						
Xingjiao	Chen						
Yan	Xu						
Jinyi	Zhou						
Ran	Tao						
Jian	Su						

Supplemental Online Content: Nonauthor Collaborators
*First name, last name, and suffix (if applicable) are required and will appear in PubMed.

*First Name and Middle Initial(s)	*Last Name	*Suffix (eg, Jr, III)	Academic Degrees	Institution	Location (city, state/province, country)	Role or Contribution, eg, chair, principal investigator	Group (if more than 1 Group listed in the byline) and/or Subgroup (eg, Steering Committee)
Xikang	Fan						
Xuejia	Chen						
Yuxiao	Huang						
Yan	Lu						
Yujie	Hua						
Li	Xing						
Shuxian	Wang						
Jianrong	Jin						
Juping	Ma						
Jiangchao	Liu						
Kaifei	Zhu						
Hongfu	Ren						
Xingfeng	Shen						
Zhenzhen	Lu						
Ge	Zhong						
Wei	Mao						
Yanxu	Zhong						
Lifang	Zhou						
Rong	Pan						
Jian	Lan						
Xiaoping	Tan						
Jinxue	Tan						
Yishan	Xie						
Liuping	Wei						
Liyuan	Zhou						
Ningyu	Chen						
Sisi	Wang						
Xianping	Wu						
Ningmei	Zhang						
Xiaofang	Chen						

Supplemental Online Content: Nonauthor Collaborators
*First name, last name, and suffix (if applicable) are required and will appear in PubMed.

***First Name and Middle Initial(s)**	***Last Name**	***Suffix (eg, Jr, III)**	Academic Degrees	Institution	Location (city, state/province, country)	Role or Contribution, eg, chair, principal investigator	Group (if more than 1 Group listed in the byline) and/or Subgroup (eg, Steering Committee)
Xiaoyu	Chang						
Zhuo	Wang						
Yujin	He						
Mingqiang	Yuan						
Ling	Wang						
Xiaofang	Chen						
Zhaodong	Wang						
Qiang	Sun						
Yang	Lin						
Faqing	Chen						
Xiaolan	Ren						
Lijun	Chang						
Feiming	Zhong						
Jianjun	Feng						
Weijie	Hu						
Xiaofang	Zhang						
Yalin	Chen						
Honghong	Wang						
Jun	Wang						
Linqi	Diao						
Zhiwei	Han						
Dengjun	Zhu						
Kai	Kang						
Shixian	Feng						
Wenjie	Yang						
Huizi	Tian						
Yali	Yan						
Bing	Han						
Li	Gao						

Supplemental Online Content: Nonauthor Collaborators

*First name, last name, and suffix (if applicable) are required and will appear in PubMed.

*First Name and Middle Initial(s)	*Last Name	*Suffix (eg, Jr, III)	Academic Degrees	Institution	Location (city, state/province, country)	Role or Contribution, eg, chair, principal investigator	Group (if more than 1 Group listed in the byline) and/or Subgroup (eg, Steering Committee)
Shaofang	Li						
Tianfang	Xing						
Wei	Tang						
Lei	Fan						
XiaoLin	Li						
Huarong	Sun						
Xiaocong	Zhao						
Ying	Li						
Chen	Hu						
Pan	He						
Xukui	Zhang						
Yuanyuan	Jin						
Lan	Luo						
Min	Yu						
Ruying	Hu						
Hao	Wang						
Weiwei	Gong						
Jieming	Zhong						
Meng	Wang						
Chunxiao	Xu						
Keqing	Gong						
Hao	Xu						
Yuan	Cao						
Kaixu	Xie						
Lingli	Chen						
Xiaomei	Tu						
Xiaojun	Li						
Junlong	Pan						
Li	Yin						
Huilin	Liu						

Supplemental Online Content: Nonauthor Collaborators
*First name, last name, and suffix (if applicable) are required and will appear in PubMed.

***First Name and Middle Initial(s)**	***Last Name**	***Suffix (eg, Jr, III)**	Academic Degrees	Institution	Location (city, state/province, country)	Role or Contribution, eg, chair, principal investigator	Group (if more than 1 Group listed in the byline) and/or Subgroup (eg, Steering Committee)
Yuan	Liu						
Lei	Yin						
Xian	Xie						
Jing	Wang						
Bo	Xiao						
Zongwei	Deng						
Yuan	Peng						
Libo	Zhang						
Chan	Qu						
Li	Deng						
Qili	Jiang						
Yanling	Chen						
