# Supplementary material for: Smoking, Exhaled Carbon Monoxide, and Risk of Parkinson Disease
Source: JAMA Neurol. 2026 Sep 8:e263037. Online ahead of print. doi: 10.1001/jamaneurol.2026.3037 (PMC13555506; doi:10.1001/jamaneurol.2026.3037)
Supplement: Supplementary file 3 [file jamaneurol-e263037-s003.pdf]

Data Sharing Statement

Bueno Lopez. Smoking, Exhaled Carbon Monoxide, and Risk of Parkinson Disease. *JAMA Neurol*. Published September 08, 2026. doi:10.1001/jamaneurol.2026.3037

Data
Data available: No

Additional Information
Explanation for why data not available: The China Kadoorie Biobank (CKB) is a global resource for the investigation of lifestyle, environmental, blood biochemical and genetic factors as determinants of common diseases. The CKB study group is committed to making the cohort data available to the scientific community in China, the UK and worldwide to advance knowledge about the causes, prevention and treatment of disease. For detailed information on what data is currently available to open access users and how to apply for it, visit: https://www.ckbiobank.org/data-access Researchers who are interested in obtaining the raw data from the China Kadoorie Biobank study that underlines this paper should contact ckbaccess@ndph.ox.ac.uk. A research proposal will be requested to ensure that any analysis is performed by bona fide researchers and - where data is not currently available to open access researchers - is restricted to the topic covered in this paper.
